# Dual Microbial Inoculation, a Game Changer? – Bacterial Biostimulants With Multifunctional Growth Promoting Traits to Mitigate Salinity Stress in Spring Mungbean

**DOI:** 10.3389/fmicb.2020.600576

**Published:** 2021-01-15

**Authors:** Kailash Chand Kumawat, Poonam Sharma, Sharon Nagpal, R. K. Gupta, Asmita Sirari, Ramakrishnan Madhavan Nair, H. Bindumadhava, Sudeep Singh

**Affiliations:** ^1^Department of Microbiology, Punjab Agricultural University, Ludhiana, India; ^2^Department of Plant Breeding and Genetics, Punjab Agricultural University, Ludhiana, India; ^3^Department of Soil Science, Punjab Agricultural University, Ludhiana, India; ^4^World Vegetable Center, South Asia, Hyderabad, India; ^5^Regional Research Station, Punjab Agricultural University, Bathinda, India

**Keywords:** ACC deaminase, anti-oxidative enzymes, *Enterococcus mundtii*, nutrient acquisition, *Rhizobium* sp.

## Abstract

Soil microbes play a vital role in improving plant growth, soil health, ameliorate biotic/abiotic stress and enhance crop productivity. The present study was aimed to investigate a coordinated effect of compatible consortium [salt tolerating *Rhizobium* and rhizobacterium with 1-aminocyclopropane-1-carboxylate (ACC) deaminase] in enhancing plant growth promoting (PGP) traits, symbiotic efficiency, nutrient acquisition, anti-oxidative enzymes, grain yield and associated profitability in spring mungbean. We identified a non-pathogenic compatible *Rhizobium* sp. LSMR-32 (MH644039.1) and *Enterococcus mundtii* LSMRS-3 (MH644178.1) from salt affected areas of Punjab, India and the same were assessed to develop consortium biofertilizer based on salt tolerance, multifarious PGP traits, antagonistic defense activities and presence of *nif*H, *acds*, *pqq*, and *ipdc* genes. Indole Acetic acid (IAA), *P*-solubilization, biofilm formation, exo-polysaccharides, siderophore, salt tolerance, ACC deaminase activities were all found highly significant in dual inoculant (LSMR-32 + LSMRS-3) treatment compared to LSMR-32 alone. Under saline soil conditions, dual inoculant showed a higher seed germination, plant height, biomass, chlorophyll content and macro and micro-nutrient uptake, than un-inoculated control. However, symbiotic (nodulation, nodule biomass and leghaemoglobin content) and soil quality parameters (phosphatase and soil dehydrogenase enzymes) increased numerically with LSMR-32 + LSMRS-3 over *Rhizobium* sp. LSMR-32 alone. Dual bacterial inoculation (LSMR-32 + LSMRS-3) increased the proline content (2.05 fold), anti-oxidative enzymes *viz*., superoxide dismutase (1.50 fold), catalase (1.43 fold) and peroxidase (3.88 folds) in contrast to control treatment. Decreased Na^+^ accumulation and increased K^+^ uptake resulted in favorable K^+^/Na^+^ ratio through ion homeostasis. Co-inoculation of *Rhizobium* sp. LSMR-32 and *Enterococcus mundtii* LSMRS-3 significantly improved the grain yield by 8.92% and led to superior B: C ratio over *Rhizobium* sp. alone under salt stress. To best of our knowledge this is perhaps the first field report from Indian soils that largely describes dual inoculation of *Rhizobium* sp. LSMR-32 and *Enterococcus mundtii* LSMRS-3 and the same can be considered as a game-changer approach to simultaneously induce salt tolerance and improve productivity in spring mungbean under saline stress conditions.

## Introduction

Improving productivity and quality of crops to feed the growing population is a major limiting factor worldwide. Producing 70% food crops for the 2.3 billion population by 2050 is a great challenge to current agriculture (Shrivastava and Kumar, 2015; GAP Report, 2018; Albdaiwi et al., 2019). Enhancing crop productivity in agro-ecological systems is intricate and greatly affected by various environmental factors in terms of agro-climatic conditions, integrated nutrient management approaches and cropping systems (Egamberdieva et al., 2019; Gupta and Pandey, 2019a). In 21^st^ century, increasing global population and lesser availability of cultivated land causing threats to sustainability due to loss of irrigation water resources, soil salinization, water and environmental pollution (Shahbaz and Ashraf, 2013). Among these, soil salinity is one of the most devastating stresses, which causes major reduction in crop productivity, quality and cultivated land area (Foyer et al., 2016; Nadeem et al., 2016; Singh and Jha, 2017). The ethylene conversion from, 1-aminocyclopropane-1-carboxylate [ACC] (immediate precursor) secreted as root exudates has been seen in all crops under biotic and abiotic stresses (Abiri et al., 2017). The stress hormone ethylene is involved in the regulation of various plant metabolic activities causing significant reduction in plant growth and development. If ethylene production is not optimized, it can result in plant senescence (Sharma et al., 2016). All over the world, soil salinity has affected more than 1000 million hectares of cultivated land (Gupta and Pandey, 2019a) causing loss of 27.3 billion US$ in crop production affecting 20 and 33% of total cultivated and irrigated agricultural lands, respectively (Qadir et al., 2014; Bhise and Dandge, 2019). Hence, it is essential to improve the salt tolerance in crops to ultimately minimize the grain yield losses.

Several techniques, including conventional breeding, gene cloning and genetic engineering have been used to improve salt tolerance in crop plants. However, such interventions often fail to alleviate stress, due to complex salinity tolerance processes and narrow germplasm genetic variability (Krishna et al., 2015; Sharma et al., 2016). Enhancing agricultural productivity through exploiting environment friendly approaches and improving irrigated land of agricultural importance is already practiced. The struggle to fight worldwide food scarcity and saline soil stress around the interest of researchers to salinity tolerance approaches (Rashid et al., 2016). Major challenge is to accelerate efficiency and sustainability of the present agriculture system affected by many abiotic and biotic stresses (Vaishnav et al., 2016). Therefore, there is an essential need to find alternative approaches that can ensure competitive grain yields, provide environmental safety, protection from phyto-pathogens as well as maintain long term ecological balance in the agro-ecological system. Use of potential indigenous salt tolerating microbial inoculants as bioenhancers/bioprotectants for enhancing agricultural sustainability is becoming a more widely accepted technique in intensive agriculture system (Gupta and Pandey, 2019a).

Salt tolerating microbes present in plant rhizosphere, rhizoplane and endosphere are proven alternatives to agro-chemicals and other conventional agronomic approaches in promoting plant growth and alleviating salt stress in plants (Etesami and Beattie, 2018; Gouda et al., 2018; Grobelak et al., 2018; Nagargade et al., 2018; Albdaiwi et al., 2019; Gupta and Pandey, 2019a). The plant growth promoting (PGP) effect of the salt tolerant plant growth promoting rhizobacteria (PGPR) is well explained by secretion of metabolites which directly induce growth. Various mechanisms have been postulated to explain how PGPR benefits the crop plants. These includes their ability to produce phyto-hormones or growth regulators such as Indole Acetic Acid (IAA), Cytokinins and Gibberellins (Albdaiwi et al., 2019), solubilization/mineralization of inorganic or organic phosphates, nutrients (zinc), biological nitrogen fixation (Etesami and Beattie, 2018) and antagonistic activities against phyto-pathogens *viz.*, iron sequestration through siderophores (Shameer and Prasad, 2018) and synthesis of HCN, antibiotics, cell wall degrading enzymes and fungicidal compounds (Singh and Jha, 2016; Kumawat et al., 2019a; Nagpal et al., 2020).

Salt tolerating rhizobacteria are known to reduce environmental stress by lowering ethylene levels through hydrolyzing 1-amino-cyclopropane-1-carboxylic acid (ACC) using ACC deaminase enzyme. ACC is the immediate precursor of hormone ethylene in plants. Various PGPR with ACC deaminase enzymes can degrade ACC to ammonia and α-ketobutyrate, thus reducing the endogenous ACC level in plants (Sharma et al., 2016; Albdaiwi et al., 2019; Nagpal et al., 2019). Under salinity stress, PGPR alter the ion homeostasis of K^+^/Na^+^ ions; accumulation of compatible solutes or osmolytes, biofilm, exo-polysaccharide production (EPS), stabilize membrane lipids and induce the transcription factors as stress response mediators (Singh and Jha, 2016; Goswami and Deka, 2020). Beneficial microbes under abiotic stress conditions activate plant antioxidant mechanisms, regulate key enzymes, such as peroxidase, catalase and superoxide dismutase (SOD) enabling the host to scavenge high amounts of reactive oxygen species (ROS) and thereby protecting it from salt toxicity (Islam et al., 2016; Singh and Jha, 2016).

Therefore, salt tolerant microbial types with ACC deaminase are known to curb salt stress caused due to higher ethylene levels. Research literature suggests that salt tolerant bacterial strains with ACC deaminase activity mitigate various biotic and abiotic stresses such as attack by phyto-pathogens, salinity, drought and flood conditions in plants (Pourbabaee et al., 2016; Ravanbakhsh et al., 2017; Ghosh et al., 2018; Albdaiwi et al., 2019; Gupta and Pandey, 2019b; Paco et al., 2020). Implementation of biofertilizer able to tolerate salt stress can function as a promising technology in terms of economic, environmental and agronomic benefits and can largely be applied to partially replace agro-chemicals known to enhance economic yield under stress conditions. Indigenous potential bacterial strains can easily acclimatize to the natural conditions and synergise the plant-microbial interactions to promote plant growth (Zahid et al., 2015).

Mungbean [*Vigna radiata* (L.) R. Wilczek var. *radiata*] is an important short duration (55–60 days) leguminous crop rich in protein, minerals (Fe and Zn) and is widely cultivated in parts of Asia, especially India due to its ability to increase soil fertility through biological nitrogen fixation (Reetha et al., 2014). The present study was thus designed with following objectives (i) isolation and identification of salt tolerating *Rhizobium* and rhizobacteria with ACC deaminase enzyme activity from salt stress conditions in spring mungbean (ii) biocompatibility assessment of potential indigenous salt tolerating *Rhizobium* strain (LSMR-32) and *Enterococcus mundtii* (LSMRS-3) for multifarious PGP traits to develop consortium biofertilizer (iii) Assessment of consortium inoculants in enhancing growth, nodulation status, antioxidant enzyme activities, nutrient acquisition, productivity and profitability/economic benefit of spring mungbean under rice-wheat cropping system.

## Materials and Methods

### Sample Collection Sites

Plant and soil samples were collected from salt affected spring mungbean fields from different sites of Regional Research Station (RRS), Bathinda (79.9455° East longitude, 30.2110° North latitude and 202 m altitude), Punjab Agricultural University (PAU), Ludhiana, Punjab, India, during April and May, 2015–2016. The region is characterized by semi-arid and very hot climate with 421 mm mean annual precipitation and temperatures above 44°C during summer (Singh et al., 2010). Five healthy spring mungbean plants (at flowering stage) were collected from salt affected areas along with the soil samples (0–15 cm depth) in the sterile plastic zip lock bags and placed in ice packs before their transfer to the Pulses Microbiology Laboratory, Department of Plant Breeding and Genetics, PAU, Ludhiana, Punjab, India. The large soil aggregates and non-rhizospheric soil was removed from plant samples. Rhizospheric soil was separated from the uprooted plants and mixed well to form one composite rhizospheric soil sample from salt stressed soil. Rhizospheric soil (0–15 cm depth) was analyzed for various chemical and physical properties. The soil texture, electrical conductivity (EC) and pH were analyzed by Hydro method (Estefan et al., 2014) and saturated paste extraction method, respectively (Bates, 1954; Richards, 1954). Organic carbon content was measured as per standard protocol (wet combustion) rapid titration method described by Walkley and Black (1934).

### Isolation and Characterization of Salt Tolerating *Rhizobium* and Rhizobacteria

To isolate *Rhizobium* and rhizobacteria from soil, plant roots were washed to get rid of the loose soil aggregates leaving behind the rhizospheric soil. Soil (10 grams) was suspended and mixed in 90 mL of sterile distilled water containing 0.5% NaCl solution. Thereafter, 10 fold serial dilutions were prepared up to 10^–7^ and 100 μL of 10^–6^ diluent was placed on different media *viz.*, Nutrient Agar (NA) for rhizobacteria (Kumawat et al., 2019a) and Congo Red Yeast Extract Mannitol Agar (CRYEMA) for *Rhizobium* (Nagpal et al., 2020) supplemented with 10% NaCl. The plates were incubated for 7 days at 28 ± 2°C and monitored regularly for colony formation (Fischer et al., 2007). Bacterial isolations were also done from nodules of spring mungbean roots washed with distilled water to remove rhizospheric soil. Nodule surface was sterilized by immersing in 70% ethanol for 2 min. followed by 2% sodium hypochlorite solution for 5 min and then washing with autoclaved distilled water (five times). Surface sterilization efficiency of the nodules was confirmed on the specific media by method described by Kumawat et al. (2019a).

Thereafter, 1 g of surface disinfected nodule was macerated aseptically in a sterile pestle mortar with 9 mL of 0.5% NaCl solution. Serial dilutions (upto 10^–7^) were prepared from macerated tissues and 0.1 mL aliquot from 10^–6^ dilution was spread on plates containing CRYEMA and NA media supplemented with 10% NaCl. The plates were incubated at 28 ± 2°C upto 7 days and observed for *Rhizobium* and rhizobacterial colonies (Ramadoss et al., 2013). All morphologically distinct colonies were screened for ACC deaminase activity on the autoclaved minimal Dworkin and Foster (DF) salt medium supplemented with 3 mM ACC instead of (NH_4_)_2_SO_4_ as sole nitrogen source (Dworkin and Foster, 1958; Penrose and Glick, 2003). Plates were incubated at 28 ± 2°C for 5 days and growth was monitored regularly. Colonies of *Rhizobium* and rhizobacteria growing on DF medium were taken as ACC deaminase producers and further purified by sub-culturing and maintained at 4°C. Commercially available dual inoculants of *Rhizobium* sp. LSMR-1 (NCBI accession no. KR072691.1) and *Stenotrophomonas maltophilia* RB-3 (NCBI accession no. KR080703.1) collected from Pulses Microbiology Laboratory, Department of Plant Breeding and Genetics, Punjab Agricultural University, Ludhina, Punjab, India were included in the study as references culture. On the basis of salt tolerance and qualitative ACC deaminase activity, *Rhizobium* sp. LSMR-32 (from nodules) and rhizobacterium LSMRS-3 (from rhizospheric soil) was selected and maintained on YEMA and Triticase Soybean Agar (TSA) medium, respectively in 3–5% glycerol and kept at −20 to −25°C for future use (Arora et al., 2014).

### Identification of Potential Salt Tolerating Bacterial Isolates From Spring Mungbean

Morphological characteristics of salt tolerating *Rhizobium* and rhizobacterial isolates was done by Gram’s staining reaction according to Bergey’s manual of Determinative Bacteriology (VIII^th^ edition) (Vincent, 1970). Isolates were physiologically and biochemically characterized by different tests such as oxidase, nitrate reduction, catalase, starch hydrolysis, IMViC [Indole, Methyl red, Voges Proskauers) and citrate utilization], sugar utilization as per carbohydrate utilization kit (Kb-009, Himedia) and Intrinsic Antibiotic Resistance (IAR) spectra to different antibiotics [*viz.* tetracycline (10 and 30 μg), erythromycin (5, 10 and 15 μg), ciprofloxaxin (1, 5, 10 and 30 μg), chloramphenicol (10, 25, 30 and 50 μg), gentamycin (10 and 50 μg), ampicillin (2, 10 and 25 μg), streptomycin (10, 25 and 300 μg), penicillin (1, 2 and 10 μg), kanamycin (5 and 10 μg) and amoxicillin (10 μg) was done according to Sarode et al. (2009). Cell motility and colony shape were observed under the Labomed LX-200 LED binocular microscope at × 100 (Kumawat et al., 2019a).

### Phyto-Pathogenicity Assay

*In vitro* pathogenicity test of selected potential indiginous salt tolerating bacteria was done by streaking on NA plates supplemented with 5% (v/v) sheep blood and incubated at 37 ± 2°C for 2–3 days. The assay was carried out in quadruplicate. Absence/presence of halo zones around the bacterial colonies was considered as negative/positive for hemolytic ability/pathogenicity for the humans.

### Bio-Compatibility Assay of Salt Tolerating *Rhizobium* sp. LSMR-32 With Rhizobacterium LSMRS-3

Selected potential strains of salt tolerating bacterial isolates, rhizobacterium LSMRS-3 containing ACC deaminase was screened for its biocompatibility with *Rhizobium* sp. LSMR-32 on Modified Succinate Agar (MSA) medium (Subramanian et al., 2015). Mutual proto-cooperation was detected between rhizobacteria LSMRS-3 and *Rhizobium* sp. LSMR-32 for growth in terms of optical density (600 nm) at 3^rd^, 6^th^ and 9^th^ day of incubation in Modified Succinate Broth (MSB) as single or combined inoculants. Further, enumeration of *Rhizobium* LSMR-32 and rhizobacterium LSMRS-3 in compatible dual inoculated broth was done by serial dilution plating technique on CRYEMA and NA media, respectively and incubated at 28 ± 2C° for 9 days in quadruplicate.

The viable population of combined inoculants was calculated by following formula and results were recorded in log cfu mL^–1^.

Log(a×b)n=loga+nlogb

Where a = mean number of specific bacterial colonies

b^*n*^ = dilution factor.

Further, LSMR-32 and LSMRS-3 (single and in combination) were assessed for multifarious PGP traits, *i.e.*, ACC deaminase, salt tolerance, drought tolerance, P and Zn – solubilization, Indole Acetic Acid (IAA), siderophore, exo-polysaccharide, biofilm formation, HCN and cell wall degrading enzymes (*viz.* β-1-4-glucanase and protease) production as per standard protocols with minor modifications (incubated at 28 ± 2°C for 5–7 days) along with commercially available cultures (LSMR-1 and RB-3).

### Quantitative Estimation of ACC Deaminase Enzyme Activity

Quantitative ACC deaminase activity of the isolates was estimated spectrophotometrically in terms of α-ketobutyrate estimated at 540 nm and compared with standard α-ketobutyrate (Sigma-Aldrich, United States) ranging between 0.1 and 1.0 mM according to method described by Shrivastava and Kumar (2013) with minor modifications (incubated at 28 ± 2°C for 4–5 days). The assay was carried out in quadruplicate. According to the standard curve, a unit of ACC deaminase activity was expressed as the amount of α-ketobutyrate produced in mMol per microgram of cellular protein/hour.

### *In vitro* Bioassay for Salinity and Drought Tolerance

#### Osmoadaption Assay

Osmoadaptation assay of salt tolerating bacterial isolates *Rhizobium* sp. LSMR-32 and rhizobacterium LSMRS-3 as single and combination treatments of was assessed for their salinity tolerance at 0, 4, 8, 12 dS m^–1^ salt concentrations as per protocol described by Zahir et al. (2010). Growth of bacterial isolates was recorded in terms of optical density (540 nm) at 28 ± 2°C for 2–3 days in Luria Bertani (LB) broth amended with different salt concentrations in quadruplicate. Bacterial isolates were also streaked on LB agar medium amended with different NaCl concentrations (@ 1, 5, 7.5, and 10%). Plates were incubated for 4–5 days at 28 ± 2°C in quadruplicate and assessed of different growth patterns according to Kaur et al. (2014).

#### Drought Tolerance

For evaluation of drought tolerance, exponential growth phase of single and dual bacterial inoculants were streaked on LB agar medium supplemented with different concentrations of Polyethylene Glycol (PEG 6000 @ 1, 5, 7.5, 10, 15 and 20%) to develop different osmotic potentials ranging from −0.045 to −0.90 MPa. Twenty percent Polyethylene Glycol (PEG 6000) was supplemented in the medium for inducing drought stress (−0.90 MPa) (Michel and Kaufmann, 1973; Ali et al., 2014). The plates were incubated for 4–5 days at 28 ± 2°C in quadruplicate and observed for different cell growth patterns of potential bacterial isolates.

### Screening of Selected Potential Salt Tolerant Bacterial Isolates for Multifarious PGP Traits

*In vitro* bioassay for auxin production as IAA equivalent was assessed for selected ACC deaminase producing (*Rhizobium* sp. LSMR-32 and *Enterococcus mundtii* LSMRS-3) bacterial isolates as single or combined treatments along with reference cultures in the presence of L-tryptophan (an immediate precursor of IAA) as per standard method described by Gordon and Weber (1951). Qualitative and quantitative phosphate solubilizing activity was measured according to method described by Jackson (1973) and Nautiyal (1999) with minor modifications (incubated at 28 ± 2°C for 15 days). Qualitative and quantitative (catecholate type) siderophore production by bacterial isolates was assessed and yellow color intensity estimated at 560 nm (Arnow, 1937; Schwyn and Neilands, 1987). Zinc solubilization efficiency using Zinc Oxide (ZnO) and Zinc phosphate (Zn_3_PO_4_) separately supplemented in Tris-minimal medium was assayed with minor modification (incubated in dark at 28 ± 2°C for 7 days) according to Kumawat et al. (2019a). The selected ACC deaminase producing bacterial isolates were also characterized for indirect multifunctional PGP traits, *i.e.*, Protease (Chaiharn et al., 2008), β-1-4-glucanase (cellulase) (Ariffin et al., 2006) and Hydrogen Cyanide (HCN) production (Bakker and Schippers, 1987). Further, potential ACC deaminase producing *Rhizobium* sp. strain (LSMR-32) and *Enterococcus mundtii* (LSMRS-3) individually as well as in combinations along with reference cultures (LSMR-1 and RB-3) were screened for exopolysaccharide production (Meneses et al., 2011) and biofilm formation (O’Toole and Kolter, 1998). All the bioassays were carried out in quadruplicate.

#### 16S rRNA Sequencing and Phylogenetic Tree Analysis of Selected Potential Salt Bacterial Isolates

The selected salt tolerating isolates were identified by partial 16S rRNA sequencing and phylogenetic tree was constructed. Genomic DNA of two potent ACC deaminase producing bacterial isolate (*Rhizobium* sp. LSMR-32 and rhizobacteria LSMRS-3) was extracted with Wizard®Genomic DNA purification kit (Promega, Madison, WI, United States). The quantity and quality of genomic DNA from bacterial isolates was analyzed by gel electrophoresis on 0.8% agarose gel and visualized by UV light under the gel documentation system. The amplification of universal 16S rRNA gene region (approximately 1500 bp) was performed in a Polymerase Chain reaction (PCR) using universal primers: Forward 8F (5′-AGAGTTTGATCCTGGCTC-3′) and reverse 1492R (5′-ACGGCTACCTTGTTACGACTT-3′) according to Giongo et al. (2010). The amplified PCR product of 16S rRNA genes was purified by using Qiagen minelute gel extraction kit (Qiagen, Thermo Fisher Scientific, United States). The final purified PCR products were sequenced from both direction by Sanger’s di-deoxynucleotide sequencing method using Bigby terminator V3 cycle sequencing kit (Applied Biosynthesis, United States) and on Applied Biosystems, 3730 automated sequencer analyzer (Applied Biosystem, United States) at Xcelris gene sequencing laboratory, Ahmedabad, India. The homology searches for the forward and reverse sequences were performed manually using Basic Local Alignment Search Tool (BLAST_*n*_ tool) and compared against sequence available in the GenBank from National Centre for Biotechnology Information (NCBI)^1^. Phylogenic analysis of 16S rRNA sequences of bacterial isolates with closely related reference sequences was carried out using Molecular Evolutionary Genetic Analysis (MEGA 6.0 software package) (Tamura et al., 2013). The sequences were aligned using multalin algorithm and output was used to construct a phylogenetic tree by calculation distance metrics for Neighbors Joining (NJ) technique with the Kimura two parameter models and a bootstrapping analysis with 1000 replicates to test robustness of internal branches between the reference sequences. The consensus sequences of ACC deaminase producing *Rhizobium* sp. LSMR-32 and *Enterococcus mundtii* LSMRS-3 obtained in our study were submitted in NCBI GenBank database and assigned the NCBI accession numbers MH644039.1 and MH644178.1, respectively.

### PCR Amplification of *acds, pqq, ipdc*, and *nif*H Genes of Selected Bacterial Strains

Amplification of the *acds* (ACC deaminase enzyme), *pqq* (Pyrroloquinoline quinine), *ipdc* (indole pyruvate decarboxylase) and *nif*H (dinitrogenase reductase) genes was performed in 20 μL of reaction mixture containing 1.0 μL (100 pM/μL) of each primer, 2 μL (30 ng/μL) of genomic DNA, 8 μL of master mix (Master mix kit containing 50 units/ml of Taq DNA polymerase, 400 μM dNTP and 3 mM MgCl_2_) (Promega, United States) and 9.0 μL of sterilized double distilled water. PCR profile for different gene specific primers is mentioned in Supplementary Table 1 with different annealing temperature. The presence and band size of specific genes from amplified PCR product was confirmed by gel electrophoresis on 1.5% agarose gel stained with Goodview dye (BR Biochem Life Science Pvt. Ltd.) and visualized under gel documentation.

#### Effect of Salt Tolerant Bacterial Strains on Growth, Nodulation Efficiency, Nutrient Acquisition, Soil Enzymes and Grain Yield Under Normal v/s Saline Soil Conditions

Field experiment were carried out continuously in the same field for three consecutive years (2017–2018 to 2019–2020) in saline soils during the spring season at Regional Research Station, Bathinda, Punjab and at Pulses Research Farm, PAU, Ludhiana, Punjab (on normal soil). The physico-chemical properties of saline and normal soils of experimental sites are described in Supplementary Table 2. The experimental plots were arranged (0.15 hectare area) in Randomized Block Design (RBD) with quadruplicate of each treatment. Seeds of recommended variety of spring mungbean (SML-668) were surface sterilized with 70% (v/v) ethanol for 1 min. followed by immersion in 2% (v/v) sodium hypochloride solution (NaClO) for 5 min. and finally washed with sterile double distilled water (4–5 times). Surface sterilized spring mungbean seeds (SML-668) were inoculated (for 30–45 min.) with bacterial inoculant suspension (10^8^–10^9^ cfu mL^–1^ in a 1:1 ratio) grown in DF salt minimal medium amended with 3 mM ACC substrate and air dried aseptically in laminar air flow. All the agronomic package of practices were followed for growing spring mungbean (Bhatti and Kaur, 2020). Observations on seed germination (%) were recorded at 10 days after sowing (DAS) and plant height, dry weight of shoot and root, chlorophyll content (Witham et al., 1971) were noticed at flowering stage (35 DAS). Symbiotic traits such as nodulation, nodule biomass, leghaemoglobin content (Wilson and Reisenauer, 1963), macronutrients accumulation *viz*. N, P and K (McKenzie and Wallace, 1954; Jackson, 1967, 1973) and micronutrients such as Na^+^, Zn, Fe, Mn, and Cu content in shoot were recorded at flowering stage (35 DAS) (Isaac and Kerber, 1971; Munns et al., 2010). Soil enzyme activities *viz.* soil dehydrogenase (Tabatabai, 1982) and phosphatase (Tabatabai and Bremner, 1969) were also registered at flowering (35 DAS) stage. Yield attributing traits such as number of seed pod^–1^, number of pod plant^–1^, 100 seed weight, seed protein content and grain yield of spring mungbean was observed at harvest (60 DAS).

#### Effect of Salt Tolerant Bacterial Strains as Single and Combined Inoculation on Proline Content and Antioxidant Enzyme Activities

##### Proline content

Proline content of salt stressed leaves was estimated by previously described standard protocol (Bates et al., 1973). One gram of fresh leaf tissues were crushed in 6 mL of 5% (w/v) sulfosalicylic acid. Centrifugation was done at 10,000 rpm for 8–10 min. Supernatant extract (1 mL) was made 2 mL with sterile distilled water and gently vortexed with Ninhydrin (2 mL). The mixture was then incubated at 100°C for 45 min. After completion of incubation, mixture was cooled and equal volume of toluene was added to the supernatant. Pink chromophore in the upper aqueous phase was used to measure color intensity at 520 nm (Elico, UV-VIS spectrophotometer). The bioassay was carried out in quadruplicate. The proline content was estimated by comparing the absorbance with a standard curve of L-proline (Sigma-Aldrich, United States).

##### Antioxidant bio-assay

Plant root samples (1.0 g) were extracted in 10 mL of 0.1 M phosphate buffer (pH 7.5) amended with 1% polyvinyl pyrrolidone (PVPP). The root extract was centrifuged for 15 min. at 10,000 rpm (4°C) and supernatant was used for antioxidant assay. SOD activity was monitored by the capacity to decrease absorbance due to color formation by nitro-blue tetrazolium (NBT) (Dhindsa et al., 1981). The reaction mixture contained 100 μL of crude enzyme extract, 0.2 mL of 200 mM methionin, 0.1 mL of 2.25 mM NBT, 0.1 mL of 3 mM EDTA, 1.5 mL of 100 mM phosphate buffer (pH 7.8), 0.1 mL of 1.5 M Na_2_CO_3_ and 0.1 mL of 2 μM riboflavin. The final volume was made 3 mL with distilled water and incubated at 28 ± 2°C under 15 W two fluorescent tubes for 10 min. to allow the development of purple color formazon and absorbance measured at 560 nm against the blank. Hundred microliter distilled water was used as blank instead of crude enzyme extract. The SOD enzyme activity was measured in terms of 50% inhibition of NBT photooxidation at 560 nm and expressed as a unit μmol O_2_ mg^–1^protein.

The peroxidase (POD) activity in the root extract was determined by the method of Onsa et al. (2004). The enzyme reaction mixture consisted of 0.1 mL of crude enzyme extract with 0.1 M phosphate buffer (pH7.0), 0.1 mM pyrogallol, 5 mM H_2_O_2_ which was incubated at 28 ± 2°C for 5–10 min. Hundred microliter of 2.5 N H_2_SO_4_ was added for turning off the reaction and indigo color intensity was measured at 420 nm (Elico UV-VIS spectrophotometer) against the blank containing water instead of enzyme extract. Catalase (CAT) enzyme activity was estimated by monitoring the decrease in absorbance of H_2_O_2_ at every 10 s. interval for 1 min. at 240 nm wavelength (Elico UV-VIS spectrophotometer) (Aebi, 1984). The reaction mixture (3 mL) consisted of 100 μL crude enzyme extract with 1.5 mL of 100 mM potassium phosphate buffer, pH 7.5), 0.1 mL of 3 mM EDTA and 0.5 mL of 0.75 mM H_2_O_2_ and volume made up to 3 mL with autoclaved distilled water. The catalase activity was calculated based on extinction coefficient of 0.04 mM^–1^ at 240 nm. All the bioassays were carried out in quadruplicate.

$$\begin{array}{c} \mathrm{Catalase activity}\left({\mathrm{Umg}}^{-1}\mathrm{protein}\right)=\frac{\Delta EM^{-1}}{0.036}\times \frac{\mathrm{volume made}}{\mathrm{volume taken}}\times \frac{1}{W} \end{array}$$

Δ*EM*^−1^ = *Change* in optical density per minute; W, weight of tissue in gram; U, μ moles H_2_O_2_ oxidized.

#### Profitability of Spring Mungbean Cultivation (Economic Return Analysis)

Gross return (Rs ha^–1^), net return (Rs ha^–1^), and benefit cost (B: C) ratio were calculated on the basis of existing cost of spring mungbean cultivation.

Gross return (Rs ha^–1^) = Grain yield (Kg ha^–1^) × pooled mean of minimum support price of spring mungbean.

Net return (Rs ha^–1^) = Gross return -Total variable cost of cultivation (Rs ha^–1^) as per treatment.

Benefit cost (B: C) ratio = Net return ÷ Total variable cost of cultivation (Rs ha^–1^) as per treatment.

Minimum support price of spring mungbean was INR 5575 quintal^–1^, INR 6975 quintal^–1^, and INR 7050 quintal^–1^ during 2017–2018, 2018–2019, and 2019–2020, respectively. Economic return was estimated by considering average price of spring mungbean for 3 years, *i.e.*, INR 6533 quintal^–1^. Total variable cost (Rs ha^–1^) was taken as the common cost per 100 kg involved for growing spring mungbean (*e.g.*, herbicide, pesticides, irrigations, field preparation, hoeing, seeds, fertilizers and labor *etc*.) along with biofertilizers as per treatment.

### Statistical Analysis

All the parameters are represented as quadruplicate of mean ± SD (standard deviation) by using Microsoft excel 2016. Further, pooled analysis of 3 years data of various growth parameters was subjected to analysis of variance (ANOVA) using SPSS (version 15.0). The significant difference between mean of single and combined inoculation treatments was analyzed with high range statistical domain (HSD) using Tukey’s tests. The treatment means were separated by the least significant difference (LSD test at *P* < 0.05).

## Results

### Isolation, Morphological, Physiological, Biochemical Characterization and Pathogenicity Test of Selected Potential Bacterial Isolates of Spring Mungbean

The healthy plants were collected from severely salt affected area of Punjab, *i.e.*, Rauli (Bathinda), Bhagsar (Bathinda) and Muktsar, Punjab, India. The soil of all the collection sites was sandy loam in texture. Soil pH, Ec and organic matter content are summarized in the Supplementary Table 3. Maximum Ec was recorded at Bhagsar (Bathinda) followed by Muktsar and Rauli and witnessed reduced plant growth as well as symbiotic efficiency.

A total of 21 *Rhizobium* and 42 rhizobacteria were isolated from nodules and rhizospheric soil on CRYEMA and NA medium supplemented with 10% NaCl. Out of total 53 bacterial isolates, *Rhizobium* sp. LSMR-32 and rhizobacterium LSMRS-3 were selected on the basis of salt tolerance (@10% NaCl concentration) and high growth on DF minimal salt medium containing ACC (Supplementary Table 4).

Selected potential bacterial isolates, *i.e.*, LSMR-32 (*Rhizobium* sp.) and LSMRS-3 (rhizobacteria) from nodule and soil of spring mungbean plants from salt stress soil, capable of utilizing ACC as N source on DF minimal salt medium were further characterized on the basis of morphological, physiological and biochemical assays. Isolate LSMR-32 exhibited rod, motile, entire shaped, white translucent, odorless, medium size, convex, mucoid and regular margin on the CRYEMA, whereas rhizobacteria LSMRS-3 produced coccus, cream, round and raised shaped, mucoid, entire margin, small and odorless colonies on NA medium amended with 10% NaCl (Supplementary Figures 1a–d). Based on Bergey’s Manual of Determinative Bacteriology, *Rhizobium* LSMR-32 was Gram negative and was positive for oxidase, catalase, indole, amylase, nitrate reduction, urease, cellulase (β-1-4-gluconase), protease (casein) and citrate utilization, but was negative for methyl red and Voges Proskauer’s test. Whereas rhizobacterium LSMRS-3 was Gram positive and indole, Voges Proskauer’s, citrate utilization, amylase, nitrate reduction, cellulase (β-1-4-gluconase) and protease (casein) tests were positive, but the isolate was found negative for urease, methyl red, catalase and oxidase tests as described in Supplementary Table 5.

Examining the carbohydrate use patterns, it was found that salt tolerant *Rhizobium* LSMR-32 and rhizobacterium LSMRS-3 utilized different carbon sources depicted in Supplementary Table 5. The IAR spectra of the *Rhizobium* LSMR-32 showed its resistance to ampicillin, streptomycin, penicillin, amoxicillin whereas sensitivity to tetracycline, erythromycin, ciprofloxacin, chloramphenicol, gentamicin and kanamycin at different concentrations (Supplementary Table 5). Similarly, antibiotic sensitivity profiling of rhizobacterium LSMRS-3 showed its sensitivity to tetracycline, gentamicin, ciprofloxacin, chloramphenicol, streptomycin, kanamycin, erythromycin and resistance to ampicillin, amoxicillin and penicillin (Supplementary Table 5 and Supplementary Figures 2a–g).

### Bio-Compatibility Interaction Assay of *Rhizobium* sp. (LSMR-32) With *Enterococcus mundtii* (LSMRS-3)

**FIGURE 1:**
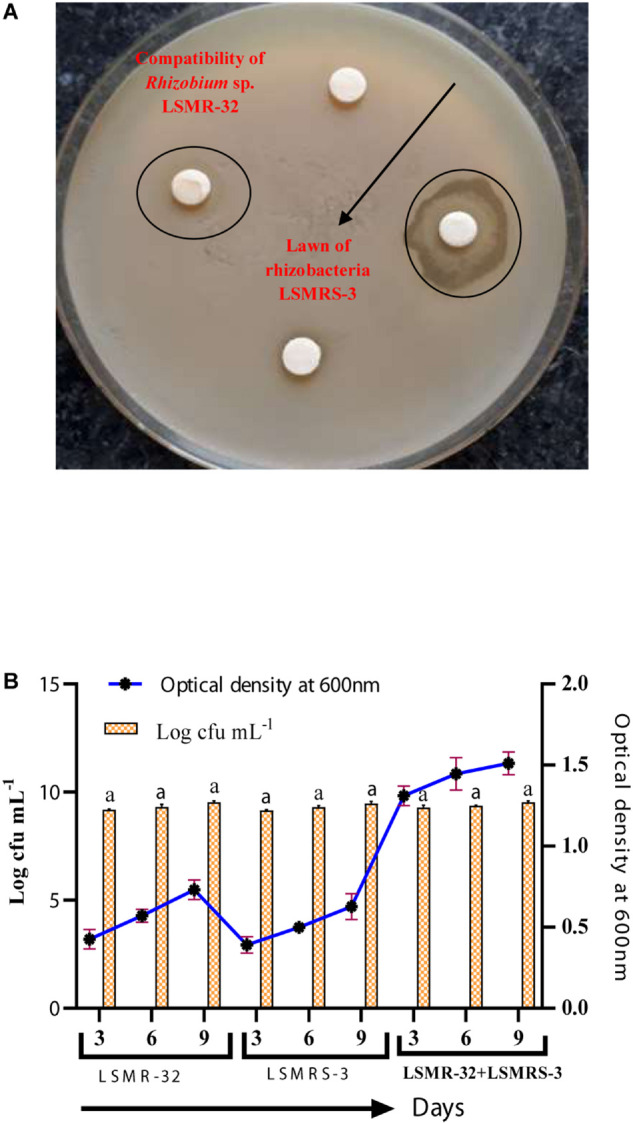
**(A)** Compatibility of *Rhizobium* sp. LSMR-32 with rhizobacterium LSMRS-3 and **(B)** Optical density at 600 nm and population density (log cfu mL^–1^) of single and dual inoculants at different intervals of time.

Salt tolerant *Rhizobium* sp. (LSMR-32) and *Enterococcus mundtii* (LSMRS-3) did not show any inhibitory effect for growth toward each other on MSA in disk plate method (Figure 1A). Mutual proto-cooperation was detected between *Enterococcus mundtii* (LSMRS-3) and *Rhizobium* sp. (LSMR-32) interms of optical density (OD) 600 nm at different intervals of time. Highest optical density was registered with LSMR-32+LSMRS-3 in comparison to single inoculant of LSMR-32 and LSMRS-3 at 9^th^ day of incubation (Figure 1B). At 9^th^ day of incubation, maximum population density as viable number of cells was noticed with combined inoculation of LSMR-32 + LSMRS-3 in contrast to monoculture inoculation of LSMR-32 and LSMRS-3 on respective media (Figure 1B).

### ACC Deaminase Enzyme Activity and *in vitro* Tolerance to Salinity and Drought

**FIGURE 2:**
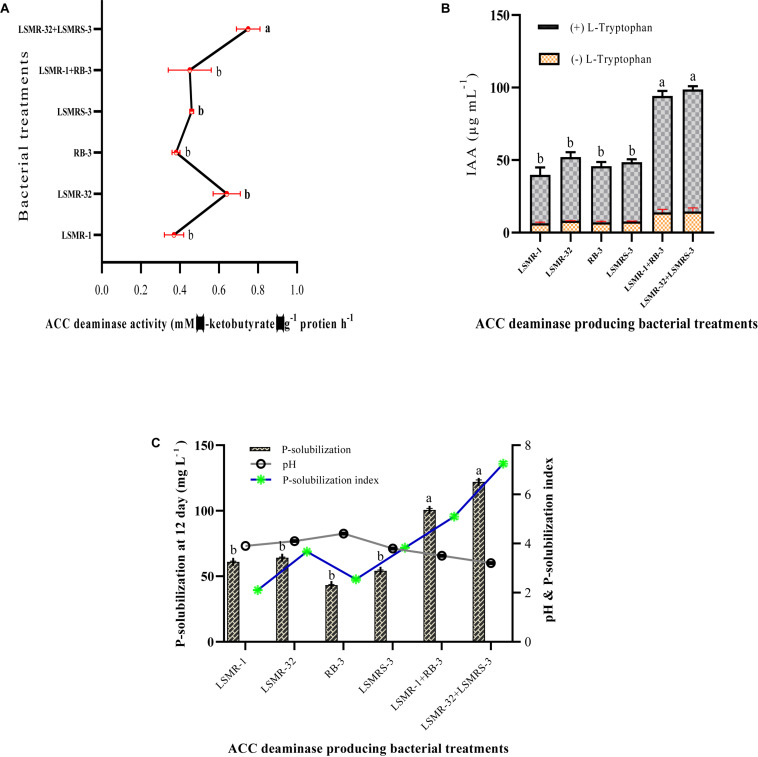
Quantitative estimation of **(A)** ACC deaminase enzyme activity, **(B)** IAA production, and **(C)** phosphate solubilization of selected potential single and dual salt tolerating bio-inoculants from spring mungbean in respective Dworkin and Foster (DF) minimal salt, LB-Trp and Pikovaskaya’s medium. Columns represent mean values while bars represent standard deviation (*n* = 3). Different letters show statistically significant different values (*P* < 0.05) from each other as evaluated from Turkey’s test.

Single as well as combined inoculations showed variation in ACC deaminase activity in the range of 0.37 ± 0.05–0.75 ± 0.06 mM α-ketobutyrate μg^–1^protein h^–1^ (Figure 2A). Combination of LSMR-32 + LSMRS-3 exhibited significantly higher ACC deaminase activity over the single inoculant *Rhizobium* sp. LSMR-32.

*Rhizobium* sp. LSMR-32 and *Enterococcus mundtii* LSMRS-3 individually as well as their combinations were assessed for their salinity tolerance at different (0–12 dS m^–1^) salinity levels and are depicted in the Supplementary Table 6. Growth response in terms of optical density at 8 dS m^–1^ salinity level ranged from 0.89 ± 0.02 – 1.88 ± 0.04 (Table 1). At 8 dS m^–1^ salinity level, combined inoculation of LSMR-32 + LSMRS-3 exhibited significantly higher growth response over single inoculants LSMR-32 and reference culture *Rhizobium* sp. LSMR-1. Further, selected potential bacterial isolates were screened for their response to different salt concentrations (1–10%) on agar plates and summarized in the Supplementary Table 6 and Table 1. Similarly, drought tolerance bioassay was carried out to assess tolerance to different concentration of PEG 6000 (1–20%) by plate assay method (Supplementary Table 6). Combination of LSMR-32 + LSMRS-3 showed highest drought tolerance in terms of growth pattern followed by monoculture of LSMR-32 and LSMRS-3 at 5% PEG6000 (−0.23 mPa). None of the isolates could tolerate high concentration of PEG6000 (20% PEG *i.e.*, −0.90 mPa) on LB agar plates.

**TABLE 1:** Multifarious PGP traits, salt and drought tolerance of selected ACC deaminase producing bacterial isolates from spring mungbean.

Treatments		LSMR-1	LSMR-32	RB-3	LSMRS-3	LSMR-1 + RB-3	LSMR-32 + LSMRS-3
**Salt and drought tolerance**							
Osmoadaptation assay @ 8 d S m^–1^		0.89 ± 0.02	1.67 ± 0.04	0.95 ± 0.05	1.51 ± 0.04	1.27 ± 0.08	1.88 ± 0.04
Salt tolerance @ 10% NaCl		+	+++	++	+++	+++	++++
lDrought tolerance @ 5% PEG 6000		+	+++	+	++	++++	++++
**Multifarious PGP traits**							
Zn solubilization efficiency (%)	ZnO	223.87	315.40	207.61	471.43	310.56	533.15
	Zn_3_(PO_4_)_2_	279.03	557.10	218.77	414.29	335.07	598.37
Siderophore production (μg ml^–1^)		142.8 ± 5.8	360.9 ± 16.5	145.4 ± 7.2	197.8 ± 12.6	152.3 ± 6.70	377.6 ± 10.1
EPS production (μg ml^–1^)		136.4 ± 13.6	212.9 ± 13.3	193.1 ± 6.7	254.7 ± 14.7	226.2 ± 3.1	257.5 ± 5.1
Biofilm formation at 12 h		0.330 ± 0.10	0.471 ± 0.02	0.429 ± 0.05	0.458 ± 0.02	0.690 ± 0.01	0.740 ± 0.05
β-1-4-gluconase (Cellulase)		+	++	+	++	++	+++
Protease (Casein)		+	+++	++	++	+++	++++
Intrinsic Antibiotic Resistance (IAR) Spectra (%)		14.8	33.3	7.2	22.2	25.8	47.8

### Multifarious PGP Traits, Molecular Characterization and Detection of *acds, pqq, ipdc, and nif* H Genes

In our study, maximum IAA production was noticed in the presence of L-tryptophan in comparison to absence of tryptophan in LB broth medium. IAA production ranged between 33.44 ± 5.07 – 84.09 ± 2.23 μg mL^–1^ with L-tryptophan after 6^th^ day of incubation in all individual and dual combinations. Combined inoculation (LSMR-32 + LSMRS-3) resulted in significantly higher IAA followed by reference inoculant LSMR-1+RB-3 with Salkovaski’s reagents (Figure 2B and Supplementary Figure 3a).

Salt tolerant *Rhizobium* and rhizobacterial isolates along with reference cultures were screened for their phosphate solubilization activity on NBRIP medium amended with 0.5% TCP as inorganic phosphorus (Supplementary Figure 3b). Yellow clear halo zone of P-solubilization was displayed around the colonies indicating P-solubilization and maximum P-solubilization index (PSI) was exhibited by dual combination of LSMR-32 + LSMRS-3 followed by LSMR-1+RB-3 (Figure 2C). Selected TCP solubilizers were further estimated quantitatively in Pikovaskaya’s broth as shown in Figure 2C. The results showed that P-solubilization potential ranged between 43.30 ± 0.33 – 122.0 ± 1.53 mg L^–1^ in all inoculants (Figure 2C). Dual combination of LSMR-32 + LSMRS-3 showed significantly higher P-solubilization followed by LSMR-1 + RB-3 at 12^th^ day of incubation. Our results indicated that dual combinations with high P-solubilization index on NBRIP agar plates showed enhanced quantitative phosphate solubilization in Pikovaskaya’s broth. The solubilization of inorganic TCP by different treatments led to significant reduction in pH (4.4 ± 0.02 – 3.2 ± 0.02) from an initial neutral pH (7.0 ± 0.02). Maximum reduction in pH was associated with LSMR-32 + LSMRS-3 followed by LSMR-1+RB-3 (Figure 2C). Negative correlation was noticed between pH and P-solubilization activity due to the production of microbial metabolites including organic acids which led to the decrease in pH of the culture broth.

Selected strains of *Rhizobium* sp. LSMR-32 and *Enterococcus mundtii* LSMRS-3 strains were found positive for multifarious PGP traits *viz*., Zn solubilization, siderophore, exo-polysaccharide, biofilm formation, β-1-4-glucanase (cellulase), protease (casein), IAR spectra and HCN production (Supplementary Figures 3c–j). The results showing multifarious PGP traits depicted in Table 1 suggest high PGP activities with combination of LSMR-32 + LSMRS-3 over single inoculants. Whereas, maximum PGP traits, *i.e.*, siderophore, zinc solubilization efficiency [with Zno and Zn_3_(PO_4_)_2_], biofilm formation and EPS was noticed with LSMR-32 + LSMRS-3 [4.63, (64.04 and 7.41), 20.95, 57.11%, respectively] treatment in contrast to *Rhizobium* sp. strain LSMR-32 alone. The IAR spectra of dual combination LSMR-32 + LSMRS-3 indicated 47.8% resistance to diverse antibiotics (Table 1). All the isolates produced β-1-4-glucanase (cellulase), protease (casein) and HCN on CMC, Skimmed milk and King’s B agar media, respectively (Table 1).

**FIGURE 3:**
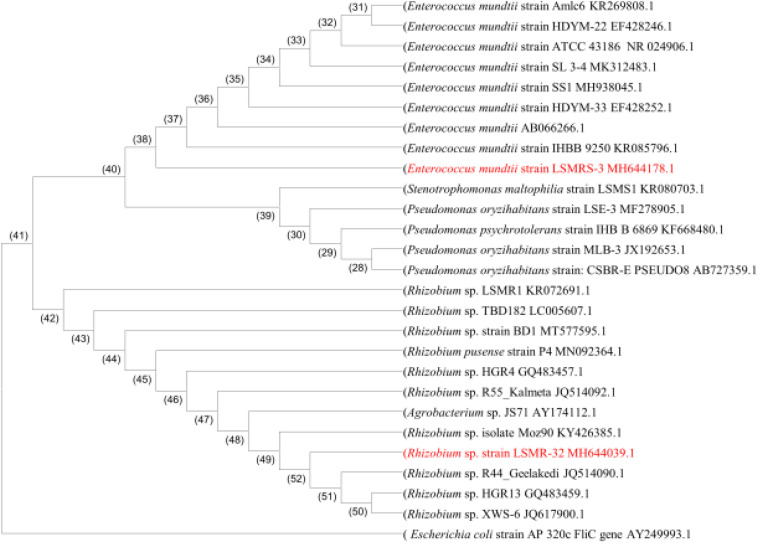
Phylogenetic tree of bacterial 16S rRNA sequence revealing *Rhizobium* sp. LSMR-32 and *Enterococcus mundtii* strain LSMRS-3 evolutionary divergence. Manually refined 16S rRNA sequences were aligned using MEGA-6 and tree was constructed using the neighbor joining method (approximately 1521 bp length fragment after refinement). GenBank accession numbers are given in parentheses.

The partial sequence analysis of 1500 bp fragment length (Supplementary Figure 4a) of 16S rRNA gene in selected salt tolerant bacterial isolates (LSMR-32 and LSMRS-3) was analyzed by nucleotide blast using blastin and clustal omega. The sequence of isolates LSMR-32 and LSMRS-3 showed similarity with *Rhizobium* sp. and *Enterococcus mundtii*, respectively and were submitted to GenBank under NCBI accession number MH644039.1 and MH644178.1, respectively. Based on ≤99% consensus sequence homology of the 16S rRNA gene sequence available in the NCBI GenBank database, strain LSMR-32 was identified as *Rhizobium* sp. and strain LSMRS-3 was assigned as *Enterococcus mundtii*. The 16S rRNA gene sequences of LSMR-32 and LSMRS-3 were aligned with sequences of other relevant PGPR of different genera and species with genera available in the GenBank database. The phylogenetic tree of selected isolates constructed using MEGA 6.0 version software revealed their proximity with other PGPR strains of respective species (Figure 3).

Genes *acds*, *pqq*, *ipdc*, and *nif*H responsible for ACC deaminase production, *P*-solubilization, IAA production and nitrogenase enzyme, respectively were PCR amplified with gene specific primers. PCR amplified product produced single bands with approximate length of 860 bp, 1000 bp, 1.7 kb and 760 bp each for *acds*, *pqq*, *ipdc* and *nif* H gene, respectively on 1.5% agarose gel stained with Goodview dye under UV light hence confirming the presence of all genes in the selected potential bacterial isolates along with the reference cultures (Supplementary Figures 4b–e). Presence of these genes suggested the capacity of selected potential salt tolerating bacterial isolates to produce ACC deaminase and nitrogenase enzyme with ability to solubilize phosphates and secrete phytohormones for improving the PGP activities and symbiotic attributes of spring mungbean under salt stress conditions.

### Effect of Salt Tolerant Bacterial Inoculants on Growth and Symbiotic Traits in Spring Mungbean

**FIGURE 4:**
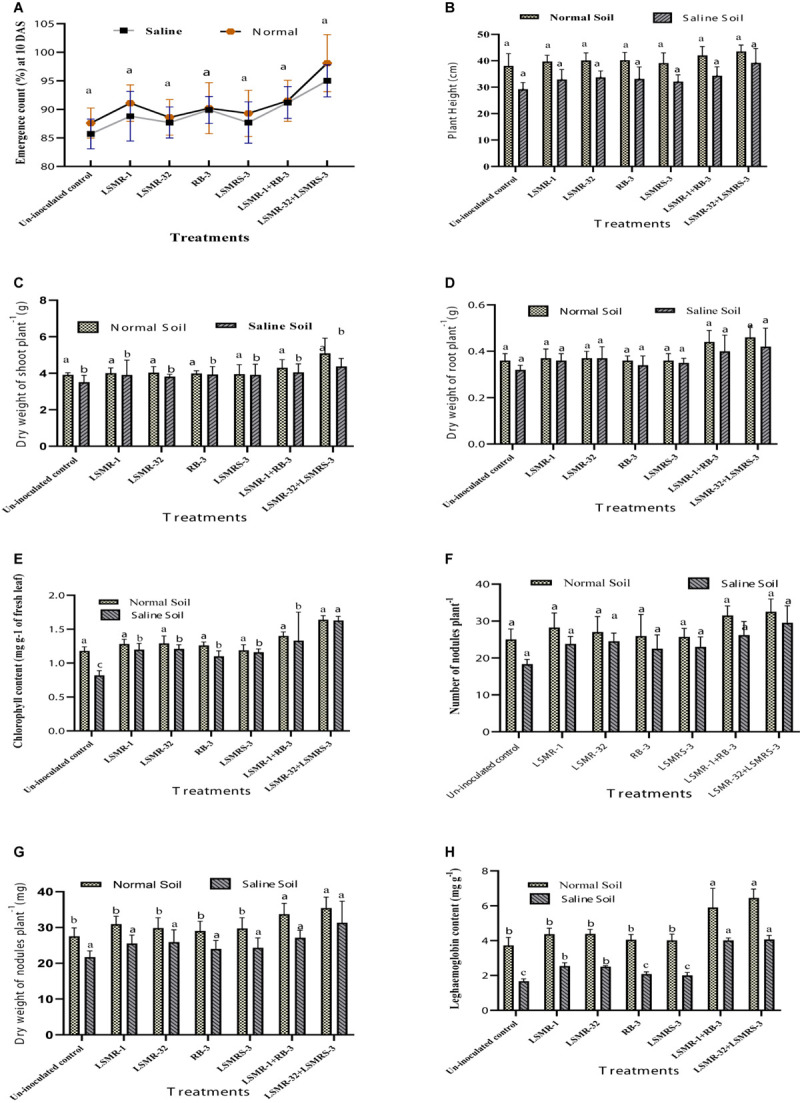
Effect of potent salt tolerant bacterial inoculants on growth and symbiotic traits: **(A)** Emergence count (%); **(B)** Plant height; **(C)** Dry weight of shoot plant^–1^; **(D)** Dry weight of root plant^–1^; **(E)** Chlorophyll content; **(F)** Number of nodule plant^–1^; **(G)** Dry weight of nodules plant^–1^ and **(H)** Leghaemoglobin content. Columns represent mean values while bars represent standard deviation (*n* = 3). Different letters show statistically significant values (*P* < 0.05) between treatments as evaluated from Duncan’s test.

The deleterious effect of salinity stress resulted in reduction in plant growth and symbiotic efficacy (*i.e.*, emergence count, plant height, dry weight of shoot and root, chlorophyll content, nodulation status, nodule biomass and leghaemoglobin content) of spring mungbean in contrast to non-stressed conditions. Pooled mean of 3 years data showed, numeric enhancement in growth and symbiotic traits with ACC deaminase producing bacterial inoculants under saline and normal soil conditions (Figures 4A–H). Numeric increment in emergence count at 10 DAS was recorded with dual combination of LSMR-32 + LSMRS-3 (10.85%) followed by recommended consortium (LSMR-1 + RB-3) (6.42%) (under normal conditions) as compared to un-inoculated control treatment (Figure 4A). Maximum improvement in plant height (Figure 4B) and dry weight of root (Figure 4D) respectively was obtained with combined inoculation of LSMR-32 + LSMRS-3 (1.34 and 1.31 folds, respectively) followed by LSMR-1 + RB-3 (1.17 and 1.25 folds, respectively) in contrast to uninoculated control under salt stress conditions. Similarly, dry weight of shoot (Figure 4C) and chlorophyll content of leaves (Figure 4E) were also significantly (*P* ≤ 0.05) enhanced by 1.24 and 1.99 times, respectively in combined inoculant LSMR-32 + LSMRS-3 in comparison to corresponding control treatment. Under normal conditions, PGP traits of spring mungbean inoculated with single as well as dual combinations did not show any significant difference over the uninoculated control treatment.

An improvement in symbiotic efficacy of plants was noticed with ACC deaminase producing bioinoculants. Combined inoculation of LSMR-32 + LSMRS-3 resulted in enrichment in nodulation and nodule biomass (20.40 and 17.68%, respectively) followed by LSMR-1 + RB-3 (6.94 and 1.88%) as compared to single LSMR-32 inoculant treatment under saline soil condition (Figures 4F,G). Significantly (*P* ≤ 0.05) higher leghaemoglobin content (62.8%) was observed with LSMR-32 + LSMRS-3 followed by LSMR-1 + RB-3 (60.8%) in contrast to single bioinoculant LSMR-32 treatment (Figure 4H). However, under normal soil conditions, nodule biomass and leghaemoglobin content (except nodulation status) in spring mungbean showed significant (*P* < 0.05) difference between the single and dual bacterial inoculants.

### Effect of Salt Tolerant Bacterial Inoculants on Macro and Micro-Nutrient Uptake of Spring Mungbean

The content of macro (N, P, and K) and micro-nutrients (Na^+^, Mn, Zn, Fe, and Cu) in the shoot of spring mungbean was negatively correlated under salt stress conditions (Table 2). Plant nutrient analysis showed that in salt stressed conditions, the N (19.14%), P (1.38%), K (0.68%), Na^+^ (21.05%), Mn (2.28%), Fe (8.08), Zn (9.49%), and Cu (31.83%) content were significantly less with combination of LSMR-32 + LSMRS-3 as compared to normal soil conditions. However, in comparison to uninoculated control treatment the inoculation of spring mungbean plants with dual combination of LSMR-32 + LSMRS-3 enhanced the uptake of N (170.54%), P (79.01%), K (20.98%), Mn (100%), Fe (49.65%), Zn (65.96%), and Cu (89.52%) content of shoot under salt stress condition. Significant reduction in Na^+^ content of shoot was noticed with combination of LSMR-32 + LSMRS-3 followed by LSMR-1 + RB-3 over uninoculated control under salt stress (Table 2). Similarly, under normal soil conditions, the inoculation of spring mungbean with dual combination of LSMR-32 + LSMRS-3 significantly increased the shoot macro and micro-nutrients over the uninoculated control treatment (Table 2).

**TABLE 2:** Effect of single *vs.* dual salt tolerant bacterial inoculants on nutrient acquisition parameters in spring mungbean.

Treatments		Un-inoculated control	LSMR-1	LSMR-32	RB-3	LSMRS-3	LSMR-1 + RB-3	LSMR 32 + LSMRS-3
**Macro-nutrient acquisition of shoot (%)**								
Total N content	Normal	1.77**^f^** ± 0.10	2.43**^de^** ± 0.06	2.77**^bcd^** ± 0.07	2.14**^ef^** ± 0.12	2.62**^cde^** ± 0.12	3.54**^a^** ± 0.17	3.61**^a^** ± 0.25
	Saline	1.12**^g^** ± 0.13	1.37**^efg^** ± 0.17	1.46**^defg^** ± 0.07	1.68**^cde^** ± 0.14	1.23**^fg^** ± 0.06	2.41**^b^** ± 0.11	3.03**^a^** ± 0.12
Total P content	Normal	0.200**^e^** ± 0.03	0.239**^cd^** ± 0.05	0.240**^bcde^** ± 0.05	0.224**^d^** ± 0.01	0.240**^bcde^** ± 0.04	0.265**^abc^** ± 0.05	0.294**^a^** ± 0.05
	Saline	0.162**^e^** ± 0.02	0.209**^cde^** ± 0.01	0.216**^bcde^** ± 0.05	0.200**^de^** ± 0.02	0.209**^cde^** ± 0.04	0.263**^abc^** ± 0.05	0.290**^a^** ± 0.03
Total K content	Normal	0.635**^c^** ± 0.06	0.698**^ab^** ± 0.04	0.695**^abc^** ± 0.05	0.675**^bc^** ± 0.06	0.688**^abc^** ± 0.07	0.723**^ab^** ± 0.05	0.743**^a^** ± 0.07
	Saline	0.610**^d^** ± 0.10	0.665**^c^** ± 0.03	0.678**^bc^** ± 0.07	0.665**^c^** ± 0.06	0.665**^c^** ± 0.03	0.705**^ab^** ± 0.07	0.738**^a^** ± 0.06
**Micro-nutrient acquisition of shoot**								
Total Na^+^ content (%)	Normal	0.083**^a^** ± 0.08	0.078**^a^** ± 0.09	0.078**^a^** ± 0.08	0.079**^a^** ± 0.06	0.078**^a^** ± 0.10	0.076**^a^** ± 0.10	0.069**^a^** ± 0.04
	Saline	0.082**^a^** ± 0.07	0.072**^ab^** ± 0.02	0.071**^ab^** ± 0.04	0.072**^ab^** ± 0.09	0.071**^ab^** ± 0.05	0.062**^ab^** ± 0.01	0.057**^b^** ± 0.01
Manganese (Mn) content (ppm)	Normal	47.66**^g^** ± 2.89	52.74**^f^** ± 4.56	55.79**^cdef^** ± 5.05	53.97**^def^** ± 4.24	53.77**^ef^** ± 2.57	63.44**^b^** ± 2.69	71.22**^a^** ± 4.14
	Saline	34.78**^g^** ± 2.32	42.49**^fg^** ± 2.30	46.83**^ef^** ± 3.36	57.38^b^**^cd^** ± 2.41	53.51**^de^** ± 1.97	58.68**^cd^** ± 2.38	69.63**^a^** ± 3.52
Iron (Fe) content (ppm)	Normal	509.29**^g^** ± 9.43	574.09**^f^** ± 9.35	612.70**^def^** ± 8.06	648.23**^*c*^** ± 11.81	605.03**^ef^** ± 7.82	719.32**^b^** ± 11.69	823.73**^a^** ± 16.57
	Saline	491.69**^g^** ± 8.24	543.03**^fg^** ± 3.99	591.00**^e^** ± 13.33	623.63**^cde^** ± 7.24	592.10**^de^** ± 8.08	663.82**^b^** ± 18.26	762.17**^a^** ± 9.40
Zinc (Zn) content (ppm)	Normal	20.93**^a^** ± 1.39	22.33**^a^** ± 3.95	23.81**^a^** ± 2.08	25.66 ± 1.75	25.20**^a^** ± 2.19	27.68**^a^** ± 2.40	34.27**^a^** ± 2.26
	Saline	18.86**^f^** ± 1.37	20.64**^ef^** ± 1.16	23.19**^def^** ± 1.56	24.07**^bcdef^** ± 0.55	23.20**^cdef^** ± 2.48	25.47**^abcde^** ± 4.17	31.30**^a^** ± 1.65
Copper (Cu) content (ppm)	Normal	4.25**^e^** ± 0.33	4.90**^de^** ± 0.18	5.63**^bcde^** ± 0.25	6.32**^abcd^** ± 0.47	5.57**^cde^** ± 0.17	6.64**^abcd^** ± 0.13	7.87**^a^** ± 0.16
	Saline	3.15**^g^** ± 0.14	3.48**^fg^** ± 0.14	3.94**^def^** ± 0.22	4.40**^cde^** ± 0.32	3.9**^ef^** ± 0.06	4.76**^bc^** ± 0.19	5.97**^a^** ± 0.25

*(Pooled mean of 3 years data; data are average values of four replication ± SD); different letters indicate significant differences based on Tukey’s HSD test at *p* < 0.05.*

### Effect of Salt Tolerant Bacterial Inoculants on Soil Enzyme Activities Under Normal vs. Saline Soil Conditions

Soil dehydrogenase, acid and alkaline phosphatase enzyme activities varied significantly among the single as well as combination of bacterial strains under normal and saline soil conditions. Under saline soil conditions, maximum percent enhancement in soil dehydrogenase enzyme activity (58.34%) was recorded with LSMR-32 + LSMRS-3 followed by LSMR-1 + RB-3 (42.26%) as compared to uninoculated control treatment. Similarly, significantly (*P* ≤ 0.05) higher acid and alkaline phosphatase enzyme activities were noticed with dual combination of LSMR-32 + LSMRS-3 (50.75 and 27.38%, respectively) followed by LSMR-1 + RB-3 (44.63 and 14.72%, respectively) in contrast to uninoculated control treatment (Table 3). Under normal soil conditions, increase in the soil enzyme activities, *i.e.*, dehydrogenase (1.57 fold), acid phosphatase (1.55 fold) and alkaline phosphatase (1.38 fold) was noticed with combined inoculation of LSMR-32 + LSMRS-3 over the uninoculated control treatment in spring mungbean (Table 3).

**TABLE 3:** Effect of single *vs.* dual salt tolerant bacterial inoculants on soil enzyme activities in spring mungbean.

Treatments	Soil quality parameters					
	Dehydrogenase activity of soil (μ g TPF g^–1^ of soil h^–1^)		Alkaline phosphatase		Acid phosphatase	
			(μ g *p*-nitrophenol ml^–1^ hr^–1^)			
			
	Normal	Saline	Normal	Saline	Normal	Saline
Un-inoculated control	49.89**^g^** ± 2.96	46.83**^a^** ± 2.84	137.74**^a^** ± 2.47	117.16**^f^** ± 2.06	54.72**^f^** ± 0.76	47.01**^f^** ± 1.34
LSMR-1	67.78**^cdef^** ± 6.39	54.17**^a^** ± 3.12	152.57**^a^** ± 2.62	130.72**^ef^** ± 5.08	66.60**^bcdef^** ± 1.06	55.03**^cdef^** ± 1.28
LSMR-32	65.36**^def^** ± 4.67	60.71**^a^** ± 3.81	147.87**^a^** ± 1.68	136.26**^def^** ± 1.13	63.80**^def^** ± 2.46	52.81**^e^** ± 2.80
RB-3	62.32**^f^** ± 2.77	57.93**^a^** ± 5.84	147.41**^a^** ± 2.02	139.34**^cde^** ± 3.24	61.48**^ef^** ± 1.18	56.91**^bcde^** ± 2.07
LSMRS-3	63.04**^ef^** ± 1.62	56.01**^a^** ± 2.36	152.21**^a^** ± 2.07	140.47**^bcde^** ± 1.94	66.57**^cdef^** ± 2.18	53.12**^def^** ± 1.48
LSMR-1+RB-3	69.76**^bcdef^** ± 4.11	66.62**^a^** ± 3.83	158.02**^a^** ± 2.34	152.50**^abcd^** ± 4.85	74.62**^abcde^** ± 1.74	67.99**^a^** ± 3.23
LSMR-32+LSMRS-3	78.47**^a^** ± 4.65	74.15**^a^** ± 3.69	175.46**^a^** ± 3.15	161.43**^a^** ± 2.87	84.83**^a^** ± 1.47	70.87**^a^** ± 2.21

*(Pooled mean of 3 years data; data are average values of four replication ± SD); different letters indicate significant differences based on Tukey’s HSD test at *p* < 0.05.*

### Effect of Salt Tolerant Bacterial Inoculants on Protein Content and Yield Attributing Traits

Under salt stress condition, high protein content and yield attributing traits, *i.e.*, number of pod plant^–1^, number of seed pod^–1^ and 100 seed weight were registered with combined inoculation of LSMR-32 + LSMRS-3 as compared to uninoculated control treatment (Table 4). Significantly (*P* ≤ 0.05) higher percent enhancement in grain yield (15.39%) was observed with combination of LSMR-32 + LSMRS-3 followed by recommended consortium LSMR-1 + RB-3 (8.39%) in contrast to un-inoculated control treatment (Table 4). Similar trend was seen in the protein content and yield parameters under normal soil condition which is summarized in Table 4. Present study did not reveal any significant difference between single and dual combination treatments for protein content and yield attributes (except 100 seed weight and grain yield) under normal soil conditions (Table 4).

**TABLE 4:** Effect of single *vs.* dual salt tolerant bacterial inoculants on protein and yield attributing traits in spring mungbean.

Treatment	Protein content of seed (%)		No. of pods plant^–1^		No. of seeds pod^–1^		100 seed weight (g)		Grain yield (Kg ha^–1^)	
	Normal	Saline	Normal	Saline	Normal	Saline	Normal	Saline	Normal	Saline
Un-inoculated control	23.65**^a^** ± 2.15	22.60**^a^** ± 2.99	22.20**^a^** ± 1.24	21.40**^a^** ± 1.43	9.50**^a^** ± 0.86	8.60**^a^** ± 1.20	4.65**^d^** ± 0.28	4.17**^a^** ± 0.64	1186**^b^** ± 109.05	942**^g^** ± 13.45
LSMR-1	23.78**^a^** ± 3.11	23.35**^a^** ± 2.27	23.60**^a^** ± 0.48	22.30**^a^** ± 2.48	10.20**^a^** ± 1.80	9.20**^a^** ± 1.21	4.85**^bcd^** ± 0.20	4.58**^a^** ± 0.12	1290**^a^** ± 57.66	991**^defg^** ± 23.64
LSMR-32	23.91**^a^** ± 4.76	23.65**^a^** ± 4.75	23.70**^a^** ± 1.15	22.60**^a^** ± 2.82	10.50**^a^** ± 0.45	9.80**^a^** ± 0.39	4.75**^cd^** ± 0.14	4.63**^a^** ± 0.46	1297**^a^** ± 41.14	998**^cdefg^** ± 32.36
RB-3	23.81**^a^** ± 2.54	23.76**^a^** ± 3.27	22.90**^a^** ± 1.78	22.10**^a^** ± 1.95	10.30**^a^** ± 0.84	9.40**^a^** ± 0.80	4.94**^abc^** ± 0.13	4.37**^a^** ± 0.40	1290**^a^** ± 23.43	978**^fg^** ± 41.62
LSMRS-3	23.98 ± 2.74	23.85**^a^** ± 4.64	22.70**^a^** ± 2.03	22.35**^a^** ± 2.90	10.40**^a^** ± 0.47	9.60**^a^** ± 0.36	4.85**^bcd^** ± 0.07	4.44**^a^** ± 0.30	1283**^a^** ± 13.53	988**^efg^** ± 10.53
LSMR-1+RB-3	24.45**^a^** ± 2.97	24.05**^a^** ± 2.92	24.10**^a^** ± 1.75	23.65**^a^** ± 3.32	10.90**^a^** ± 0.43	10.10**^a^** ± 0.49	5.01**^ab^** ± 0.16	4.89**^a^** ± 0.35	1334**^a^** ± 47.32	1021**^bcdef^** ± 24.75
LSMR-32+LSMRS-3	25.10**^a^** ± 3.88	24.78**^a^** ± 0.73	24.60**^a^** ± 3.97	23.85**^a^** ± 1.55	11.10**^a^** ± 1.44	10.80**^a^** ± 1.50	5.10**^a^** ± 0.12	5.08**^a^** ± 0.19	1357**^a^** ± 70.38	1087**^a^** ± 60.80

*(Pooled mean of 3 years data; data are average values of four replication ± SD); different letters indicate significant differences based on Tukey’s HSD test at *p* < 0.05.*

### Effect of Salt Tolerant Bacterial Inoculants on Osmolyte Accumulation (Proline) and Anti-oxidative Enzyme Activities

**FIGURE 5:**
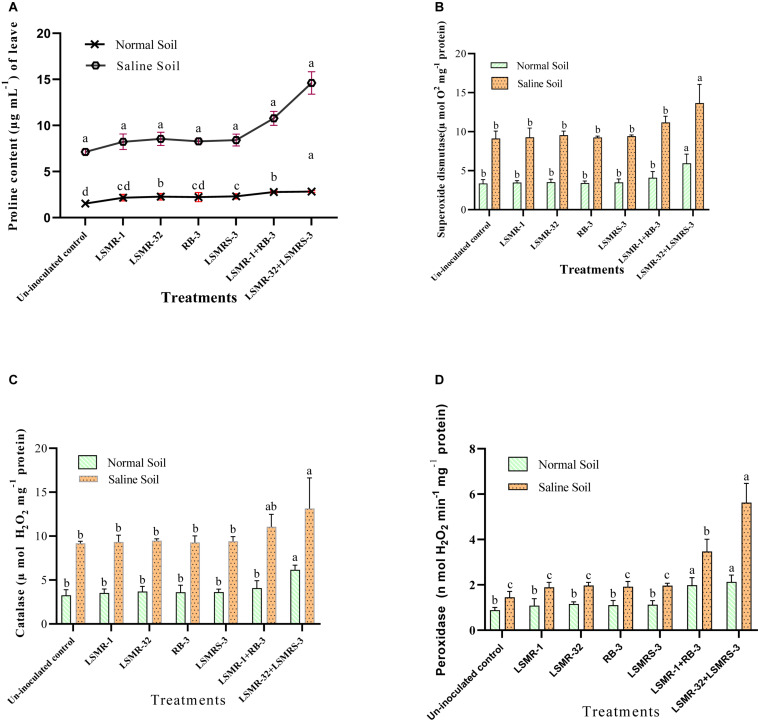
Effect of potent salt tolerant bacterial inoculants on proline content and anti-oxidant enzyme activities under stress conditions **(A)** Proline content; **(B)** Superoxide dismutase; **(C)** Catalase; and **(D)** Peroxidase enzyme activities. Columns represent mean values while bars represent standard deviation (*n* = 3). Different letters show statistically significant different values (*P* < 0.05) between treatments as evaluated from Duncan’s test.

In our investigation, single and dual combinations increased the proline content under both normal and saline stress conditions. Co-inoculation of LSMR-32 + LSMRS-3 resulted in remarkable (2.05 fold) increase in the leaf proline content of spring mungbean followed by LSMR-1 + RB-3 (1.51 fold) over uninoculated control treatment under salt stress condition. Whereas, under normal conditions, the single as well as dual combination treatments, enhanced the leaf proline content by 1.42–1.86 folds in contrast to uninoculated control treatment (Figure 5A).

Significant difference in the anti-oxidative enzyme activities was observed between inoculated and uninoculated treatments under salinity stress condition. Significantly high SOD enzyme activity under salt stress was recorded in the spring mungbean plants inoculated with LSMR-32 + LSMRS-3 (43.08%) followed by LSMR-1 + RB-3 (17.19%) in contrast to single inoculant LSMR-32 (Figure 5B). Similarly, dual combination showed significant increase in the enzyme activities such as CAT (43.13%) and POX (287.58%) as compared to uninoculated control spring mungbean plants under saline stress condition (Figures 5C,D). However, under normal conditions, the bioinoculant treatments (single or dual combination) increased SOD, CAT and POX anti-oxidative enzyme activities (77.31, 88.65 and 142.04%, respectively) as compared to uninoculated control treatment.

### Economic Return (Profitability) of Spring Mungbean Cultivation

In our study, economic return of spring mungbean cultivation was high in the normal soil as compared to saline soil due to reduction in grain yield under stressed condition (Table 5). Highest gross return and net return were obtained with combined inoculation of LSMR-32 + LSMRS-3 over the uninoculated control treatment under saline soil conditions. Maximum B: C ratio (1.87) and additional profits (9,372.85 Rs ha^–1^) were calculated with combined application of *Rhizobium* sp. LSMR-32 + *Enterococcus mundtii* strain (LSMRS-3) as compared to un-inoculated control treatment under saline soil conditions. Similar results were seen in gross return, net return and B:C ratio under normal soil conditions which are summarized in Table 5.

**TABLE 5:** Benefit: cost ratio of potential salt tolerating bacterial inoculants application in spring mungbean.

Treatment	Grain yield (Kg ha^–1^)		Gross return (Rs ha^–1^)^a^		Net return (Rs ha^–1^)^b^		B : C ratio		Additional income over control (Rs ha^–1^)	
	Normal	Saline	Normal	Saline	Normal	Saline	Normal	Saline	Normal	Saline
Un-inoculated control	1186.0**^b^**	942.0**^g^**	77,481.38	61,540.86	52,830.38	36,889.86	2.14	1.50	-	-
LSMR-1	1290.0**^a^**	991.0**^defg^**	84,275.70	64,742.03	59,549.70	40,016.03	2.41	1.62	6,719.32	3,126.17
LSMR-32	1297.0**^a^**	998.0**^cdefg^**	84,733.01	65,199.34	60,007.01	40,473.34	2.43	1.64	7,176.63	3,583.48
RB-3	1290.0**^a^**	978.0**^fg^**	84,275.70	63,892.74	59,549.70	39,166.74	2.41	1.58	6,719.32	2,276.88
LSMRS-3	1283.0**^a^**	988.0**^efg^**	83,818.39	64,546.04	59,092.39	39,820.04	2.40	1.61	6,262.01	2,930.18
LSMR-1 + RB-3	1334.0**^a^**	1021.0**^bcdef^**	87,150.22	66,701.93	62,399.22	41,950.93	2.52	1.69	9,568.84	5,061.07
LSMR-32 + LSMRS-3	1357.0**^a^**	1087.0**^a^**	88,652.81	71,013.71	63,901.81	46,262.71	2.58	1.87	11,071.43	9,372.85

*Cost of cultivation of un-inoculated control = Rs. 24,651/-*

*Cost of cultivation of single culture inoculants = Rs. 24,726/-*

*Cost of cultivation of dual culture inoculants = Rs. 24,751/-*

*1 US$ = 75.82 Rs.*

*^(a)^Market price of spring mungbean = Rs. 6533/- quintal.*

*^(b)^Cost of microbial consortium @Rs. 40 per packet (for one acre).*

## Discussion

Soil salinization is one of the key limiting factors for agricultural production in arid and semiarid tropics. Diverse array of salt tolerating PGPR, form an integral part of soil microbiota that interact with legume plants and help in mitigating the harmful impact of salt stress, improving the growth and symbiotic efficiency. These salt tolerating bacteria are known to have multifunctional PGP traits and stress tolerance that facilitate the plants to survive under inhibitory levels of salt stress (Ali and Kim, 2018; Etesami and Beattie, 2018; Maxton et al., 2018; Nascimento et al., 2018; Saleem et al., 2018; Zhang et al., 2018). Therefore, the role of salt tolerating bacterial isolates is enormously considerable in the current agricultural system, affected by many biotic and abiotic stresses. In the last decade, several beneficial microbes belonging to different taxonomic groups such as *Rhizobium*, *Bacillus*, *Azospirillum*, *Pseudomonas*, *Azotobacter*, *Pantoea*, *Burkholderia*, *Paenibacillus*, *Serratia*, *Variovorax*, *Sphingobacterium*, *Enterobacter*, *Enterococcus*, *Stenotrophomonas*, *Alcaligenes* and *Ochrobactrum etc*. have been studied showing their favorable results on plant growth and for inducing salt stress tolerance in different crops (wheat, chickpea, alfalfa, soybean, mungbean, groundnut and tomato *etc*.) (Panwar et al., 2016; Egamberdieva et al., 2017; Chandra et al., 2018; Saikia et al., 2018; Ansari et al., 2019; Gupta and Pandey, 2019a; Khan and Bano, 2019; Vaishnav et al., 2019, 2020; Goswami and Deka, 2020; Nagpal et al., 2020).

In the present investigation, salt tolerating strains of LSMR-32 and LSMRS-3 had significant multifarious PGP traits and salt tolerance proving potential their as effective biostimulators. IAA is one of the most important plant growth hormone synthesized by 80% of rhizospheric microflora which affect the endogenous level of IAA in the plant (Pandey et al., 2019). IAA alters the root architecture promoting the formation of lateral and adventitious roots that improve the water use and nutrient uptake efficiency of plants under stress conditions (Saikia et al., 2018; Gupta and Pandey, 2019a). Endogenous plant IAA along with that produced by salt tolerating microbes enhance the secretion of plant root exudates that serve as energy sources for root associated growth promoting bacterial isolates, improving their early colonization efficiency (Etesami et al., 2015; Chandra et al., 2018).

The IAA acts as a transcription factor for ACC synthesis, which is the immediate precursor for ethylene stimulated ACC deaminase enzyme in rhizobacteria (Abd-Allah et al., 2017; Egamberdieva et al., 2018). Therefore, ACC deaminase enzyme activity and IAA production by rhizobacteria are considered as most important PGP attributes under stress conditions (Saikia et al., 2018; Albdaiwi et al., 2019). Several strains of genus, *i.e., Rhizobium*, *Bradyrhizobium*, *Nostoc*, *Leclercia*, *Azotobacter*, *Enterococcus*, *Stenotrophomonas*, *Rahnella*, *Azospirillum*, *Pseudomonas*, and *Arthrobactor etc.* were reported to produce IAA under salt stress conditions (Majeed et al., 2015; Zahid et al., 2015; Sharma et al., 2016; Verma et al., 2018; Kumawat et al., 2019a). Kumawat et al. (2019a) documented maximum IAA production with dual inoculants of *Bradyrhizobium* sp. LSBR-3 + *L. adecarboxylata* LSE-1 and enhanced growth, symbiotic efficiency and stress tolerance in soybean due to ACC and IAA producing capability of rhizobacteria. According to Lee et al. (2015) indole acetic acid and gibberellin secreting *Enterococcus faecium* strain LKE-1 enhanced plant length and biomass through endogenous secondary metabolite regulation in rice cultivars. Salt tolerating microbial diversity can tolerate high salt (NaCl) concentrations because of their capability to break immediate precursor of ethylene, *i.e.*, ACC into α-ketobutyrate and ammonia and accumulate compatible osmolytes to maintain intracellular osmotic balance under various abiotic and biotic stresses (Albdaiwi et al., 2019; Pandey et al., 2019; Vaishnav et al., 2020).

Phosphorus (P) functions as the second most crucial macronutrient after N having role in multiple plant metabolic processes such as rootstock development, flower pollination, N_2_ fixation and resistance against many phytopathogens. The abundant phosphorous (organic and inorganic) present in soils can not be utilized because plants absorb only monobasic (H_2_PO_4_^–^) and dibasic (HPO_4_^–2^) forms of phosphorus (Kumawat et al., 2019a). Phosphorus solubilizing bacteria (PSB) convert different forms of phosphorus into plant accessible forms. Dual inoculant LSMR-32 + LSMRS-3 treatment released significant amount of PO_4_^–3^ from inorganic Tri-calcium phosphate (TCP) in Pikovaskaya’s broth as compared to single inoculants. This activity could influence P uptake of shoot enhancing growth, nodulation and N_2_ fixing efficiency. *P*-solubilization by salt tolerating bacterial isolates is accompanied by a drop in pH of the medium, indicating production of organic acids (gluconic acid, oxalic acid, and citric acid) in the medium for solubilizing insoluble P forms and mineralizing organic forms by phosphatases and phytases (Chen et al., 2016; Paul and Sinha, 2017; Wei et al., 2018; Gupta and Pandey, 2019a; Kumawat et al., 2019a; Nagpal et al., 2019; Goswami and Deka, 2020). Salt tolerating bacterial isolates produced phosphatase enzyme aiding release of *P*-ions from *P*-mineral reservoir by H^+^ substitution for cation bound to phosphate (Mahdi et al., 2011). The results of present study are in accordance with numerous published reports on phosphate solubilization by species of *Paenibacillus*, *Aneurinibacillus*, *Halomonas*, *Halobacillus*, *Enterobacter*, *Bradyrhizobium*, *Rhizobium*, *Bacillus*, *Acinetobacter*, *Ochrobactrum* and *Cladosporium etc.* (Desale et al., 2014; Korir et al., 2017; Singh et al., 2017; Enebe and Babalola, 2018; Numan et al., 2018; Zhao et al., 2018; Vaishnav et al., 2019). Two salt tolerating PGPR strains closely related to *Enterococcus faecium* and *Pantoea dispersa* were able to solubilize significant amounts of phosphate with positive effect on mungbean growth under salt stress conditions (Panwar et al., 2016).

Zn is considered as an essential micronutrient for growth and biological nitrogen fixation in legumes. Selected potential strains had the Zn solubilizing ability to solubilize ZnO and Zn_3_(PO_4_)_2_ on Tris minimal medium. Iron occurs abundantly in ferric (Fe^+3^) form in the aerobic environment as hydroxides and oxy-hydroxides rendering it inaccessible to both plants and rhizobacteria (Kumawat et al., 2019a). Low molecular mass iron chelating compounds (siderophores) produced by rhizospheric microbes can bind to most of the iron available in the rhizosphere with very high acidity, inhibiting the proliferation of phytopathogens due to lack of available iron. Application of zinc solubilizers and siderophore producers is an environment friendly and sustainable approach over agrochemicals by satisfying the biological availability of zinc and iron to plants, hence contributing in plant growth and nutritional status of legume and cereal crops (Panwar et al., 2016; Kotasthane et al., 2017; Priyanka et al., 2017; Vaishnav et al., 2019; Goswami and Deka, 2020). In present investigation, salt tolerating bacterial strains (LSMR-32 and LSMRS-3) produced cell wall degrading enzymes (β-1-4-glucanase and protease), HCN and showed antibiotic resistance spectra, signifying their capacity for competition against the phytopathogen for nutrition, early colonization sites on root tips for better establishment in the rhizosphere and activating cellular defense mechanisms under stressful conditions. The production of cell wall lytic enzymes (chitinase and protease) and other antagonistic substances by salt tolerating PGPR against pathogen lead to leakage of cell contents and ultimately collapse of the evading pathogen since cell wall is responsible for maintaining the cellular integrity (Choudhary et al., 2015; Saikia et al., 2018; Bhise and Dandge, 2019; Egamberdieva et al., 2019; Nagpal et al., 2019, 2020).

Salt tolerating microbial diversity had ability to produce exopolysaccharides and biofilm during environmental stress act as a contributing factor toward the plant adjustment to salt induced osmotic shocks. The polysaccharide molecules and biofilms are produced by microbes in bid to protect themselves from desiccation causing microbial aggregation. Beneficial microbes are known to reduce the Na^+^ ion toxicity and abundance of toxic ions in rhizosphere by aggregating in mass of cell at both living and non-living surfaces present in the root environment contributing indirectly to stress tolerance (Chakraborty et al., 2016; Kasim et al., 2016; Qin et al., 2016; Naseem et al., 2018; Khan and Bano, 2019). Some salt tolerating bacterial isolates, *i.e., Planomicrobium* sp., *Rhizobium* sp., *Mesorhizobium* sp., *Bacillus* sp., *Bradyrhizobium* sp., *Pseudomonas* sp., *Marinobacter* sp., *Halomonas* sp., *Planococcus* sp., *G. diazotrophicus*, and *Enterobacter* sp., *etc*. are known to produce exopolysaccharides as well as biofilms in legumes and cereals for early colonization and adjusting plant under abiotic (salt and drought) stress conditions (Qurashi and Sabri, 2012; Egamberdieva et al., 2016; Kasim et al., 2016; Yang et al., 2016; Enebe and Babalola, 2018; Atouei et al., 2019; Chu et al., 2019; Khan and Bano, 2019). Biofilm aided by EPS production enhance resistance of PGPR to biotic and abiotic stress (Sandhya and Ali, 2015) but the role of EPS in tolerance mechanisms need more extensive investigations (Ilangumaran and Smith, 2017).

Using 16S rRNA, blast similarity searches and phylogenetic tree analysis, it was established that salt tolerating bacterial isolates in our research, belonged to *Rhizobium* sp. (LSMR-32) and *Enterococcus mundtii* (LSMRS-3), respectively. The results of present study are comparable with several previous researches who observed the most important taxonomic genera of PGPR as *Leclercia*, *Pseudomonas*, *Pantoea*, *Paenibacillus*, *Aneurinibacillus*, *Oceanobacillus*, *Halomonas*, *Enterococcus*, *Stenotrophomonas*, *Klebsiella*, *Ochrobacterum*, *Enterobacter*, *Clostridium*, *Arthrobacter*, *Micrococcus*, *Aavobacterium*, *Azospirillum*, *Rhizobium*, *Azotobacter* and *Sphingobacterium etc.* (Zahid et al., 2015; Panwar et al., 2016; Singh and Jha, 2016; Egamberdieva et al., 2017; Sreevidya and Gopalakrishnan, 2017; Gupta and Pandey, 2019a; Kumawat et al., 2019a,b; Pandey et al., 2019; Vaishnav et al., 2020). In the present study, *Rhizobium* sp. strain LSMR-32 and *Enterococcus mundtii* strain LSMRS-3 produced an amplified fragment of *nif*H, *acds*, *ipdc*, and *pqq* genes with specific primers. These genes (*nif*H, *acds, ipdc* and *pqq*) are involved in multifunctional PGP traits and enzyme activities such as nitrogen fixation, ACC deaminase, IAA production and *P*-solubilization in salt tolerating rhizobacteria improving growth as well as plant nutrition under normal and stress conditions.

Nitrogenase encoding *nif*H gene in *Azospirillum* sp. from the rhizosphere of wheat has a highly conserved Fe protein for nitrogenase and used as a marker gene for biological nitrogen fixation (Ayyaz et al., 2016). Abdel-Salam et al. (2013) reported *B. licheniformis* strain having *pqq* gene with expected amplified PCR product of 1202 bp for efficient phosphate solubilization. According to Gang et al. (2017) *Klebsiella* SGM81 strain showed the presence of *ipdc* gene and confirmed bacterial production of IAA with improvement in PGP traits of *D. caryophyllus*. In addition to *acds* gene, various strains of *Mesorhizobium*, *Rhizobium*, *Sinorhizobium*, *Microbacterium*, *Klebsiella*, *Enterococcus*, and *Pseudomonas etc*. are under the transcriptional control of *nif*A promoter responsible for activating the transcription of nitrogen fixation genes, promoting plant growth and nodulation by lowering ethylene levels under abiotic stress conditions (Shrivastava and Kumar, 2013; Glick, 2014; Khosravi et al., 2014; Chandra et al., 2018). Improved shoot and root length and biomass and relative water content were observed with *Klebsiella* sp. strain IG 3 in oat (*Avena sativa*) at NaCl stress (100 mM) with higher expression of *acds* (ACC deaminase activity) and *ipdc* (IAA production) genes in *Klebsiella* (Sapre et al., 2018).

Field experiment conducted under normal and saline soil conditions in present study reported symbiotic tripartite interaction of *Rhizobium* sp. LSMR-32, *Enterococcus mundtii* strain LSMRS-3 and host plant spring mungbean. Synergistic interaction between the microorganisms is a universal phenomenon in microbial ecosystems via augmenting PGP traits due to proto-cooperation with salt tolerating bacteria (Gupta and Pandey, 2019a; Samaddar et al., 2019). Our results illustrated that the coinoculation of *Rhizobium* sp. LSMR-32 and *Enterococcus mundtii* LSMRS-3 had higher potential to improve plant growth, symbiotic traits, nutrient acquisition, soil enzyme activities and yield attributing traits ultimately mitigating the harmful effects of salinity.

High number of bacterial cells in dual inoculant results in better PGP activities and symbiotic efficacy under abiotic and biotic stress conditions (Barra et al., 2016; Panwar et al., 2016; Grobelak et al., 2018; Saikia et al., 2018). Similar reports suggested that application of salt tolerating rhizobacteria enhance soil water-plant-relationship, alter phytohormone signaling, nutrient solubilization and trigger several other mechanisms that work in an integrated fashion to enhance salt and drought stress tolerance in plants (Forni et al., 2017; Vaishnav et al., 2019). Present investigation is similar with Mahmood et al. (2016) who demonstrated combined application of *Enterobacter cloacae* and *Bacillus drentensis* with 2 Kg Si ha^–1^ resulted in enhancement of mungbean physiology, growth and yield under salt stress conditions. Ahmad et al. (2011) revealed that under axenic condition, co-inoculation of *Pseudomonas* sp. (MK1, M20 and M25) and *Rhizobium* sp. (M1, M6 and M9) with ACC deaminase activity, significantly increased plant growth promotion as well as symbiotic parameters as compared to uninoculated control treatment at 6 dS m^–1^ salinity level in mungbean. Similarly, Shaharoona et al. (2006) reported that coinoculation of ACCD possessing PGPR with *Bradyrhizobium* sp. improved symbiotic traits in mungbean by lowering ethylene production as compared to *Bradyrhizobium* sp. alone. Trdan et al. (2019) reported that dual inoculants *Pseudomonas fluorescens* and *Azospirillum brasilense* enhanced shoot height, fresh and dry weight of shoot, yield and antioxidant enzyme activities in potato as compared to uninoculated control plant under abiotic stress condition. Tahir et al. (2019) also reported that rhizobacterial strains BN-5 and MD-23 secreting, ACC deaminase, IAA, *P*-solubilization, biofilm formation and exo-polysaccharide (EPS) production boosted the grain yield and quality of maize under salt as well as drought stress conditions. Present investigation is well coherent with Chandra et al. (2019) who reported three ACC deaminase containing bacterial strains (*Pseudomonas palleroniana* DPB 16, *Pseudomonas* sp. UW 4, and *Variovorax paradoxus* RAA3) enhancing growth, nutrient uptake, osmolytes as well as antioxidant enzyme activities and grain yield of wheat under salt stress conditions in contrast to uninoculated control treatment. Priming of salt tolerating *Pseudomonas knackmussii* MLR-6 to *Arabidopsis thaliana* enhanced growth and reduced oxidative damage due to salt stress as compared to uninoculated control plants (Rabhi et al., 2018). Similarly, Wang et al. (2018) reported that biopriming of pepper seedling with *Bacillus* sp. promoted the growth in terms of enhanced fresh and dry weight, shoot and root length under saline conditions.

The salt tolerating dual inoculant of LSMR-32 and LSMRS-3 besides improving root architecture and promoting higher nodulation efficiency also showed elevated nutrient (N, P, K, Mn, Zn, Fe and Cu) content in spring mungbean shoot over single LSMR-32 and uninoculated control treatment. Enhancement of nutrient availability in plant as well as soil by rhizobacteria could be due to regulation of phytohormone production, solubilization of nutrients, siderophore for iron seizure and enlarged lateral root surface area by the various biological mechanisms of plant microbe interaction under saline stress conditions (Shabayev, 2015; Sathya et al., 2017; Solanki et al., 2017; Omara et al., 2017; Egamberdieva et al., 2018). The improved macro and micro-nutrient content with dual inoculation in present research is closely correlated with Safronova et al. (2012) who demonstrated combined inoculation of *Mesorhizobium loti* and *V. paradoxus* 5C-2, possessing ACC deaminase activity having synergistic and additive effects on nodulation, root growth and nutrient acquisition (N, P, Ca, Mn, Zn and Cu) in shoot of *L. edulis* and *L. ornithopodioides*. Similarly, Rana et al. (2015) observed an improvement of 13–16% in Fe, Zn, Cu, and Mn concentration with co-inoculation of *Providencia* sp. PR-3+*B. diminuta* PR-7 + *O. anthropi* PR-10 in rice.

Significant reduction in shoot Na^+^ and increment in K^+^ was observed with dual inoculant (LSMR-32 + LSMRS-3) application under saline stress conditions. Under stressful conditions, excessive Na^+^ ions compete with K^+^ ions leading to nutrition disproportion and metabolic disorder thus negatively affecting the crops. ACC deaminase producing microbes can encourage growth of plants by altering selectivity of ions and maintaining higher K^+^ concentration as compared to Na^+^ (Panwar et al., 2016). The decreased Na^+^ accumulation and high K^+^/Na^+^ ratio by the salt tolerating rhizobacteria can be attributed to the bacterial biofilm and exopolysaccharides which bind cations (especially Na^+^) in roots, thus preventing their movement to survive the high salt concentration (Islam et al., 2016). Present illustrations are well supported by Singh and Jha (2017) who observed that inoculation of *Stenotrophomonas maltophilia* strain SBP-9 significantly decreased the accumulation of Na^+^ by 25–32% along with enhanced K^+^ uptake by 20–28% in both shoot and root of wheat grown under salinity stress. Similarly, Panwar et al. (2016) demonstrated significant decrease in Na^+^ accumulation with dual inoculation of *Pantoea dispersa* and *Enterococcus faecium* over the uninoculated control in *Vigna radiata* under salt stress condition.

Soil enzyme activities indicate the number of living microbial diversity in the plant rhizosphere. They play an important role in catalyzing metabolic reactions associated with the biomass decomposition as well as hydrolysis of organic polyphosphates and could be involved in the enhancement of plant nutrient acquisition from soil (Marinkovic et al., 2016; Siczek and Lipiec, 2016; El-Nohrawy and Omara, 2017; Bargaz et al., 2018). Significant higher soil enzymes activities were obtained with dual combination of LSMR-32 + LSMRS-3 as compared to uninoculated control treatment. Results are well coherent with earlier finding of Islam et al. (2016) where significant increase in soil dehydrogenase and phosphate enzyme activities was registered in *Vigna radiata* with *B. cereus* strain Pb-25 as compared to control treatment under saline soil condition.

Salt stress is associated with oxidative pressure overproducing ROS (OH^–^, O_2_^–^ and H_2_O_2_), harmful for plants (Panwar et al., 2016). ROS plays a significant role as signaling molecules at low concentration, but at higher level they cause oxidative damage and affect membranes, lipids, nucleic acids and proteins (Del Rio, 2015). Several reports have confirmed that the ability of plant cell and microbes to mitigate the effects of oxidative burst occurs by improving the accumulation of osmolytes and antioxidant enzymes (Egamberdieva et al., 2017; Singh and Jha, 2017; Saikia et al., 2018; Khan and Bano, 2019; Khanna et al., 2019; Rajput et al., 2019; Vaishnav et al., 2019; Zahir et al., 2019; Nagpal et al., 2020). Our study illustrated that dual inoculants of LSMR-32 + LSMRS-3 reduced osmotic and oxidative stress in spring mungbean plants by inducing accumulation of proline and antioxidant enzymes (CAT, POX and SOD) in roots, exposed to salt stress. Beneficial salt tolerating microorganisms can overcome harmful effects of salinity by maintaining a favorable ratio of K^+^/Na^+^ ions amenable for plant growth under high salt concentrations. Accumulation of compatible solutes, stabilizing membrane lipids, maintenance of redox potential, free radical scavenging by antioxidant enzymes, binding of toxic metals and induction of transcription factors are some of the mechanisms used by bacteria to suppress salt stress conditions (Singh and Jha, 2017; Albdaiwi et al., 2019; Ansari et al., 2019; Gupta and Pandey, 2019b; Goswami and Deka, 2020; Vaishnav et al., 2020).

In this study, increased protein content as well as yield attributing traits was recorded in *Vigna radiata* with dual inoculation of *Rhizobium* sp. LSMR-32 and *Enterococcus mundtii* LSMRS-3 which might be due to synergistic potential as well as reduced osmotic and oxidative stress through ROS production under salt stress conditions. Ahmad et al. (2013) demonstrated that combined inoculation of *P. fluorescence* (MK20) and *Rhizobium phaseoli* (mg6), showed synergistic effects and improved yield by 62% over control treatment in mungbean under saline soil conditions. Similarly, Aamir et al. (2013) documented that co-inoculation of *Rhizobium* (A2) and PGPR containing ACC deaminase improved plant growth, symbiotic traits and yield of mungbean by reducing the stress induced by ethylene production. Ahmad et al. (2014) also demonstrated that coinoculation of *Rhizobium* and rhizobacterium containing ACC deaminase induced salt tolerance and led to increased plant growth and yield attributes in mungbean. Another study performed by Tittabutr et al. (2013) also revealed that combined inoculation of mungbean with *Bradyrhizobium* sp. and PGPB containing stress induced ACC deaminase alleviated effect of different environmental stress conditions. Dual inoculation of *Mesorhizobium ciceri* IC-53 + *B. subtilis* NUU4 (Egamberdieva et al., 2017) and *Rhizobium* sp. RH4 + PGPR strain P5 (Ullah et al., 2016) enhanced plant growth, symbiotic efficacy and yield attributing traits in chickpea over the uninoculated control treatment under salt stress condition. In our study we obtained maximum B: C ratio (1.87) and net return (24.40%) with salt tolerating dual inoculant of LSMR-32 + LSMRS-3 in comparison to the control treatment in spring mungbean under salt stress condition. Overall, it can be concluded that use of indigenous salt tolerating *Rhizobium* sp. LSMR-32 and *Enterococcus mundtii* LSMRS-3 in spring mungbean have more potential to adapt and reduce ethylene levels in plants, thus alleviating the harmful effects of salt stress. In the context of enhancing concern of providing sufficient food and other environmental issues, dual combination of *Rhizobium* sp. LSMR-32 and *Enterococcus mundtii* LSMRS-3 can be used as a novel consortium biofertilizer/bioprotectant minimizing requisite of agrochemicals in spring mungbean in salt affected Indo-Gangetic alluvial plains of India.

## Conclusion

To the best of our knowledge, till date, ours is perhaps the first field report from Indian soils describing the beneficial effect of dual bio-inoculant, *Rhizobium* sp. LSMR-32 and *E. mundtii* LSMRS-3, especially in spring mungbean under highly unfavorable salt stress conditions. The dual bio-inoculant was found highly effective in terms of enhancing grain yield, higher B: C ratio (1.87), which no doubt helps farmers to realize additional incomes. Our results provide a convincing evidence (as per our own confirmative *in vitro* and *in vivo* assays) that the dual bio-inoculant mitigates salt stress by altering phytohormone levels, accelerate production of antioxidant enzymes, osmolytes, reducing sodium uptake and regulating functional metabolic and physiological pathways. Nonetheless, it is important to develop new strategies and we assume our current research output perhaps provide an impetus, for more specific and efficient salt tolerating microbes that can be employed as a proxy, for saline and drought regions under Trans-Gangetic Agro-climatic belts of India, for enhancing productivity and profitability of spring mungbean crop. It is indicative that bio-inoculants have a greater potential in increasing food security and resource use efficiency.

## Data Availability Statement

The original contributions presented in the study are included in the article/Supplementary Material, further inquiries can be directed to the corresponding author/s.

## Author Contributions

KK performed the experiments, and collected, analyzed, and interpreted the data. KK and SN prepared and edited the manuscript. PS conceptualized the research work. RG helped in executing the nutrient content of plant samples and edited the manuscript. AS supervised the molecular analysis section of the work. RN and HB supported research activity and manuscript editing and reviewing. SS supervised the field experiment.

## Conflict of Interest

The authors declare that the research was conducted in the absence of any commercial or financial relationships that could be construed as a potential conflict of interest.

## Footnotes

1 https://www.ncbi.nlm.nih.gov/
