# Supplementary material for: Dual Microbial Inoculation, a Game Changer? – Bacterial Biostimulants With Multifunctional Growth Promoting Traits to Mitigate Salinity Stress in Spring Mungbean
Source: Front Microbiol. 2021 Jan 15;11:600576. doi: 10.3389/fmicb.2020.600576 (PMC7874087; doi:10.3389/fmicb.2020.600576)
Supplement: Supplementary file 1 [file Data_Sheet_1.docx]

**Table: S1**

**Gene specific primers (*acds, pqq, ipdc* and *nif*H) and their PCR profiles for selected bacterial isolates**

| **Sr. No.** | **genes name** | **Primers** | **PCR profile** | **References** |
| --- | --- | --- | --- | --- |
| 1 | *acds* (ACC deaminase enzyme) | *acds*(F) 5’-*GGCAACGGTCGACATCTAGGC*-3’  *acds*(R) 5’-*GGCTTGCCATTCAGCTAGG*-3’ | Initial denaturation for 1.30 min at 94°C, 35 cycles of 1.0 min at 94°C, 50 s, annealing at 49.9°C and 1.30 min, primer extension at 72°C and final extension step for 5 min at 72°C. | (Shrivastava and Kumar, 2013). |
| 2 | *pqq* (Pyrroloquinoline quinine) | *pqq* (F) 5’-*TGCATTTCCGCTACGATACA*-3’  *pqq* (R) 5’-*ACCGTAATACAAGGAAGCTGAA*-3’ | Initial denaturation step for 1.30 min. at 94°C, followed by 35 amplification cycles of denaturation for 1.0 min. at 94°C primer annealing at 51.9°C for 45 s, primer extension for 1.30 min. at 72°C and a final extension step for 7 min at 72°C. | (Abdel-Salam et al., 2013). |
| 3 | *ipdc* (indole pyruvate decarboxylase) | *ipdc*(F) 5’*TGATATCGCGTGGCGTTTGCCTGGTA*-3’  *ipdc* (R) 5’*GCGGATTTTCCCGGCGGTGTTCGTCG*-3’ | Initial denaturation step foe 1.30 min. at 94°C, followed by 35 cycles of 1.0 min denaturation at 94°C, 45 s, primer annealing at 63.8°C, 1.30 min primer extension at 72°C and final extension step for 7 min at 72°C. | (Jha et al., 2012) |
| 4 | *nif*H (dinitrogenase reductase) | *nif*H (F) 5’-*TGCGATCCCCGAAAGGCCGGACTC*-3’  *nif*H (R)5’-ATCGGCCATCATTCTCAGCCGGA-3’ | Initial denaturation for 1.30 min. at 94°C, 35 cycles of 1.0 min denaturation at 94°C, 50 s. *nif*H gene primer annealing at 62.2°C, primer extension for 1.30 min at 72°C and a final extension step for 5 min at 72°C. | (Kumawat et al., 2019a). |

**Table: S2**

**Physico-chemical properties of soils on experimental block at RRS, Bathinda (Saline) and Pulses Research Farm, PAU, Ludhiana (normal) conditions**

| **Soil characteristics** | **Saline soil (Bathinda)** | **Normal soil (PAU, Ludhiana)** |
| --- | --- | --- |
| Soil type | Sandy loamy | Sandy loamy |
| pH | 8.35±0.02 | 7.65±0.02 |
| EC | 0.42-0.53 | 0.27-0.30 |
| Organic matter (%) | 0.82-1.16 | 0.22-0.40 |
| Available N (Kg ha^-1^) | 124.10-168.00 | 127.35-134.40 |
| Available P (Kg ha^-1^) | 17.5-21.5 | 16.2-29.9 |
| Available K (Kg ha^-1^) | 220-610 | 151.22-179.23 |
| Available Fe (mg Kg^-1^) | 10.55-13.02 | 13.70-15.11 |
| Available Cu (mg Kg^-1^) | 0.58-1.34 | 0.54-0.89 |
| Available Zn (mg Kg^-1^) | 2.44-3.45 | 2.08-2.52 |
| Available Mn (mg Kg^-1^) | 6.42-8.75 | 7.13-9.98 |
| Bulk density (mg m^3^) | 1.56±0.02 | 1.62±0.46 |

**Table: S3**

**Site description, soil characteristics and growth stages for spring mungbean samples used in present study**

| Locations | Collection dates | Location characteristics | | | Soil characteristics | | | | Growth stage |
| --- | --- | --- | --- | --- | --- | --- | --- | --- | --- |
|  |  | Latitude | Longitude | Elevation | Soil texture | EC  (dS m^-1^) | pH | Organic matter |  |
| Rauli, Bathinda, Punjab | 12 April 2015 | 31.0657°N | 75.4556°E | -240 M | Sandy loam | 0.46±0.06 | 8.73±0.02 | 0.98±0.02 | Flowering stages |
| Muktsar, Punjab |  | 30.4762°N | 74.5122°E | -198 M | Sandy loam | 0.41±0.04 | 8.24±0.02 | 0.86±0.05 |  |
| Bhagsar, Bathinda, Punjab |  | 30.2110°N | 74.9455°E | -195 M | Sandy loam | 0.52±0.08 | 8.67±0.02 | 1.14±0.10 |  |

**Table: S4**

**Salt tolerance and ACC deaminase activity of selected bacterial isolates**

| Selected bacterial isolates | NaCl concentration levels | | | | | | ACC deaminase activity on DF medium supplemented with 3 mM ACC as N substrate |
| --- | --- | --- | --- | --- | --- | --- | --- |
|  | 1% | 2.5% | 5% | 7.5% | 10% | 15% |  |
| *Rhizobium* sp. strain LSMR-32 | ++ | ++ | +++ | +++ | ++++ | ++ | +++++ |
| *Enterococcus mundtii* LSMRS-3 | ++ | ++ | ++ | +++ | +++++ | +++ | +++++ |

+ indicate the presence of growth; ++ = low; +++ = medium; ++++ = high and +++++ = very high

**Table: S5**

**Phenotypic, biochemical and physiological characteristics of selected ACC deaminase producing bacterial isolates**

| **Characteristics** | | ***Rhizobium* sp. strain LSMR-32** | ***Enterococcus mundtii* LSMRS-3** |
| --- | --- | --- | --- |
| **Phenotypic characteristics** | | | |
| Cell Shape | | Rod | Coccus |
| Motility | | Motile | Motile |
| Colony Shape | | Entire | Round |
| Elevation | | Convex | Raised |
| Texture | | Mucoid | Mucoid |
| Margin | | Regular | Entire |
| Colour | | White Translucent | Cream |
| Odour | | Odourless | Odourless |
| Size | | Medium | Small |
| Pigmentations | | None | None |
| **Biochemical characteristics** | | | |
| Gram’s Reaction | | - | + |
| Oxidase | | + | - |
| Catalase | | + | - |
| Indole | | + | + |
| Methyl Red | | - | - |
| Voges Proskauer | | - | + |
| Urease | | + | - |
| Amylase | | + | + |
| Nitrate Reduction | | + | + |
| Cellulase | | + | + |
| Protease (Casein) | | + | + |
| Citrate utilization | | + | + |
| **Physiological characteristics** | | | |
| Mannitol | | + | + |
| Inositol | | + | - |
| Lactose | | + | + |
| Cellobiose | | + | + |
| Sucrose | | + | + |
| Sorbitol | | + | + |
| Starch | | + | + |
| Xylose | | + | + |
| Mannose | | + | + |
| Sorbose | | + | + |
| Xylitol | | - | - |
| Rhamnose | | + | + |
| Sodium Gluconate | | + | + |
| L-Arabinose | | + | + |
| Trehalose | | + | + |
| Raffinose | | + | + |
| Galactose | | + | + |
| Fructose | | + | + |
| Maltose | | + | + |
| **Intrinsic Antibiotic Resistance (IAR) assay** | | | |
| Antibiotics | Concentration (µ disc^-1^) |  |  |
| Tetracycline | 10 | S(.09) | S(1.9) |
|  | 30 | S(1.8) | S(2.0) |
| Erythromycin | 5 | S(1.2) | S(2.7) |
|  | 10 | S(1.4) | S(3.1) |
|  | 15 | S(1.8) | S(3.2) |
| Ciprofloxaxin | 1 | S(2.2) | S(2.8) |
|  | 5 | S(2.4) | S(3.0) |
|  | 10 | S(2.5) | S(3.1) |
|  | 30 | S(3.3) | S(3.2) |
| Chloraphenicol | 10 | S(1.5) | S(2.3) |
|  | 25 | S(1.7) | S(2.4) |
|  | 30 | S(2.0) | S(2.5) |
|  | 50 | S(2.1) | S(2.5) |
| Gentamycin | 10 | S(1.3) | S(2.3) |
|  | 50 | S(2.4) | S(2.3) |
| Ampicillin | 2 | R | R |
|  | 10 | R | R |
|  | 25 | S(1.2) | R |
| Streptomycin | 10 | R | S(2.1) |
|  | 25 | R | S(2.3) |
|  | 300 | R | S(2.5) |
| Penicillin | 1 | R | R |
|  | 2 | R | R |
|  | 10 | R | R |
| Kanamycin | 5 | S(1.6) | S(1.1) |
|  | 10 | S(2.2) | S(1.9 |
| Amoxicillin | 10 | R | R |

**+ = Positive; - = Negative; S = Susceptible (Zone of inhibition in centimeter); R = Resistance**

**Table S6**

***Invitro*Drought and Osmotolerance bio-assay of selected potential ACC deaminase producing bacterial isolates**

| Bacterial Isolates | Osmo-adaptation Assay | | | | Drought Tolerance Assay | | | | | |
| --- | --- | --- | --- | --- | --- | --- | --- | --- | --- | --- |
|  | Salinity Level (d S m^-1^) | | | | Concentration of PEG 6000 | | | | | |
|  | 0 | 4 | 8 | 12 | 1% | 5% | 7.5% | 10% | 15% | 20% |
| *Rhizobium* sp. strain LSMR-32 | 1.19±0.04 | 1.23±0.12 | 1.67±0.04 | 0.52±0.02 | ++ | ++++ | ++ | ++ | + | + |
| *Enterococcus mundtii* LSMRS-3 | 1.17±0.03 | 1.48±0.02 | 1.51±0.04 | 1.09±0.09 | + | ++++ | ++ | + | + | + |

| **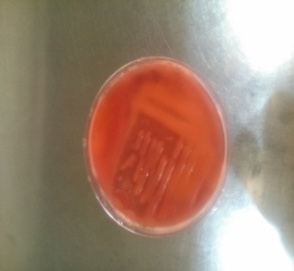**  **a** | **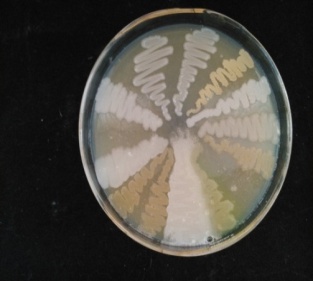**  **b** |
| --- | --- |
| **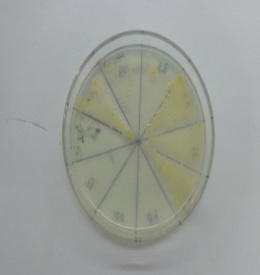**  **c** | **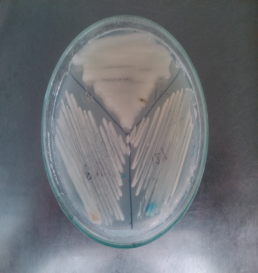**  **d** |

**Supplementary Figure 1.**(a) *Rhizobium* on CRYEMA medium (b) Rhizobacterial diversity on Triticase Soybean Agar (TSA) medium (c) Salt tolerance (d) ACC deaminase activity on Dworkin & Foster (DF) medium.

| **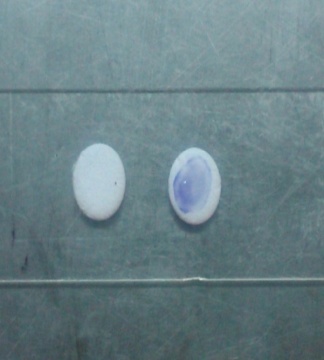**  **a** | **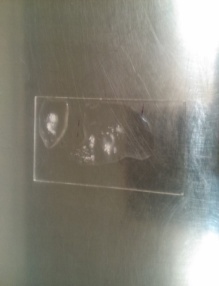**  **b** |
| --- | --- |
| **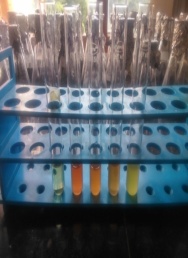**  **c** | **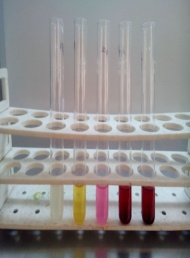**  **d** |
| **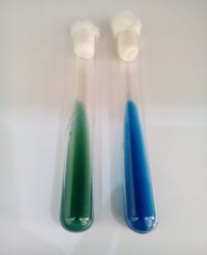**  **e** | **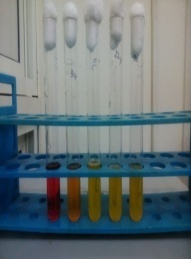**  **f** |
| **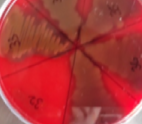**  **g** | |

**Supplementary Figure 2.**Physiological and bio-chemical tests (a) oxidase (b) catalase (c) Voges Proskauer’s (d) nitrate reduction (e) citrate utilization (f) carbohydrate utilization & (g) Pathogenicity test

| **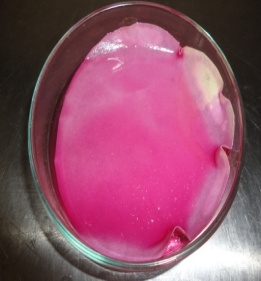 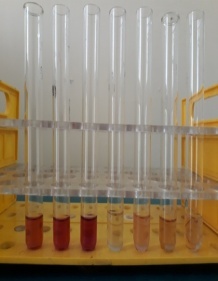**  Quantitative  Qualitative  **(a)** |
| --- |
| **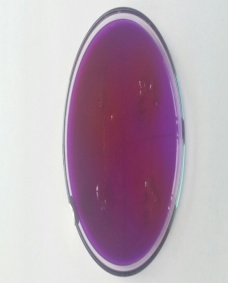 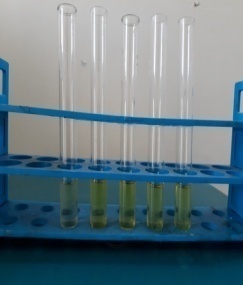**  Quantitative  Qualitative  **(b)** |
| **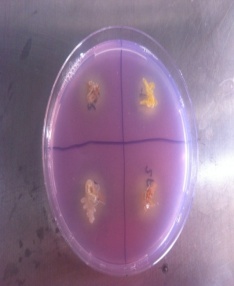 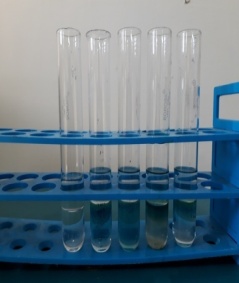**  Quantitative  Qualitative  **(c)** |

**Contd…………………..**

| **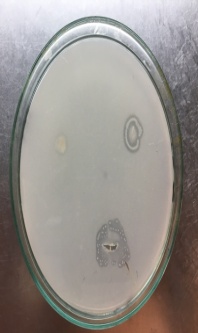**  **d** | **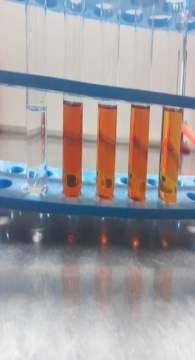**  **e** |
| --- | --- |
| **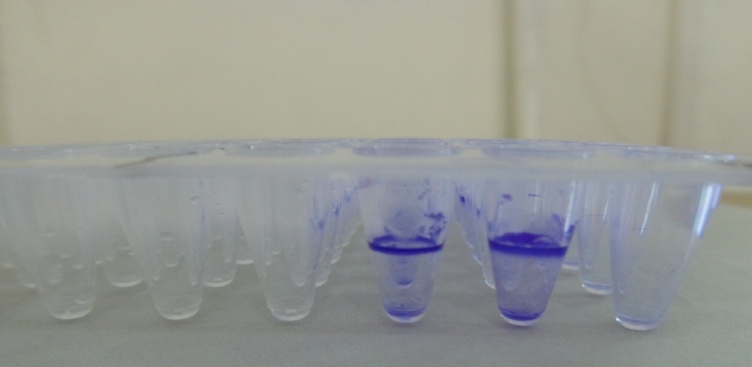**  **f** | **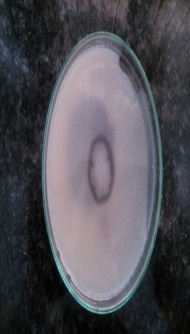**  **g** |
| **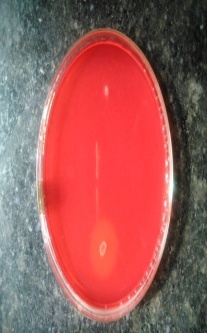**  **h** | **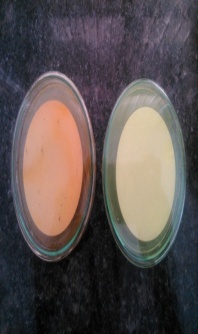**  **i** |
| **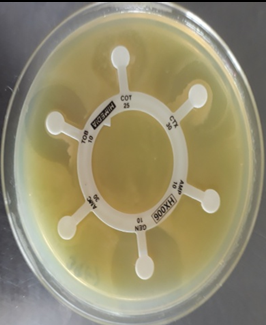**  **j** | |

**Supplementary Figure 3.**Multifunctional PGP traits (a) IAA (b) P- solubilization (c) Siderophore production (d) Zn- solubilization (e) Exo-polysaccharide production (f) Bio-film formation (g) Protease (h) Callulase (i) HCN production (j) Intrinsic Antibiotic Resistance (IAR) spectra

**
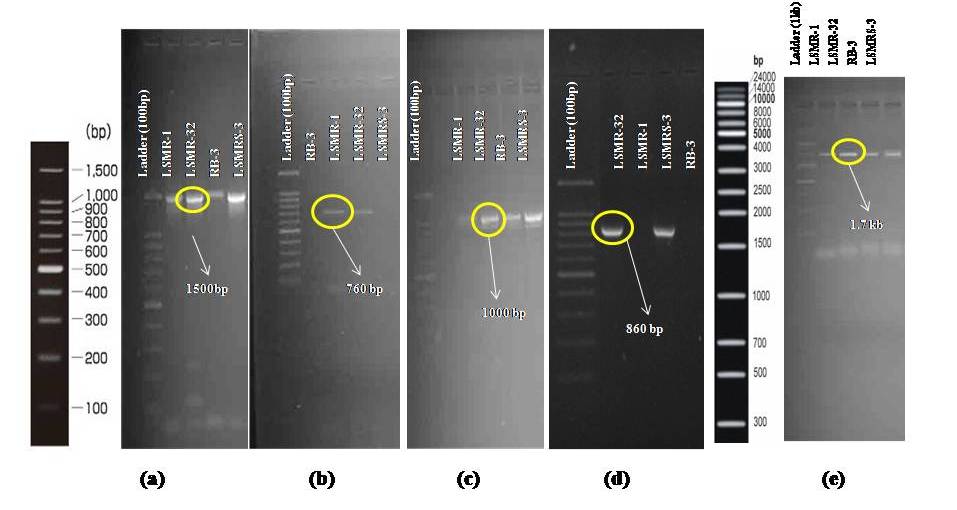
**

**Supplementary Figure 4.** PCR amplification of (a) 16 S rRNA gene (b) *nif* H gene (c) *pqq* gene (d) *acds* gene and (e) *ipdc* gene regions, Lane-1 show ladder; Lane-2 strain LSMR-1- *Rhizobium* sp.; Lane -3 LSMR-32- salt tolerating *Rhizobium* sp.; Lane-4 RB-3- *Stenotrophomonas maltophilia* and Lane -5 LSMRS-3- *Enterococcus mundtii*
